# Informality in the time of COVID-19 in Latin America: Implications and policy options

**DOI:** 10.1371/journal.pone.0261277

**Published:** 2021-12-16

**Authors:** Ivonne Acevedo, Francesca Castellani, Giulia Lotti, Miguel Székely

**Affiliations:** 1 Center for Educational and Social Studies, Mexico City, Mexico; 2 Office of Strategic Planning and Development Effectiveness, Inter-American Development Bank, Washington, District of Columbia, United States of America; University of South Florida, UNITED STATES

## Abstract

This paper analyzes the dynamics of the labor market in Latin America during the COVID-19 pandemic. After a decade of a virtuous circle of growth with the creation of formal jobs, the pandemic has had an considerable impact on the region’s labor market, generating an unparalleled increase in the proportion of the inactive population, considerable reductions in informality, and, in contrast, smaller fluctuations in formal jobs. In this context, the formal sector, given its lower flexibility, became a "social safety net" that preserved the stability of employment and wages. Based on the findings presented in this paper, it is projected that, starting in 2021, informality will grow to levels higher than those of the pre-COVID-19 era–with 7.56 million additional informal jobs–as a result of the population returning to the labor market to compensate for the declines in incomes. According to the simulations presented, postponing or forgiving income tax payments and social security contributions conditional on the generation of formal jobs could reduce the growth of informality by 50 to 75 percent. Achieving educational improvements has the potential to reduce it by 50 percent.

## Introduction

Since the onset of the COVID-19 pandemic in Latin America in early March 2020, most countries in the region implemented containment actions that have included confinement measures, mobility restrictions, and partial or total closures of economic activity, among other actions. A few studies document the types of general policies introduced in developing countries [1–3]. This has had serious consequences in the region, including declines in gross domestic product (GDP) of 7.4 percent in 2020, according to projections by the International Monetary Fund [4]. The declines vary from 1.6 percent in Paraguay to as much as 12.9 percent in Peru [5]. The International Labour Organization (ILO) estimates that in 2020 approximately 39 million people in Latin America lost their jobs [6].

Since before the pandemic Latin America has had levels of labor informality above 50 percent on average, implying that large sectors of the population were exposed to and unprotected from vulnerabilities in the face of unexpected fluctuations in the labor market. For this reason, the pandemic crisis is expected to generate high welfare costs in the region.

In previous economic crises, informality functioned as a buffer, absorbing the outflow from the formal sector and thus limiting increases in the unemployment rate [7]. However, it is likely that informal jobs will be particularly affected in the context of COVID-19 for several reasons. Personal services, which account for a considerable proportion of informality, carry higher health contagion risks because they involve interactions with other individuals for whom it is not possible to verify exposure to the virus. Informality is specifically characterized by the lack of health insurance, so attention to contagion risks may be weaker, leading to longer recovery times (and inactivity) or less effective care due to the saturation of public services. Even in cases where government authorities have implemented economic support mechanisms to cushion the drop in employment, the informal population, which is outside tax and other public registries, is more difficult to identify and locate, and therefore unlikely to benefit from active policies. As for formal companies, the obligation of severance payments and the uncertainty about the costs and benefits of altering production plans make them less flexible to adjust to the changing circumstances [8].

This paper analyzes informality during the COVID-19 pandemic in Latin America by examining the dynamics of the labor market in 2020 using the data from household and employment surveys. The paper looks at the possible evolution of informality in the years ahead and identifies the potential effect of different policies to limit its expansion. Although the COVID-19 crisis has already been the subject of several studies focused on different dimensions of social welfare in Latin America [1, 2, 9–12], the consequences on labor informality have been studied to a lesser extent [13–15]. For the analysis, formal employment refers to workers with access to social security, while informal employment refers to workers who do not contribute to social security. In this respect, data from sources used for this paper show that most of the informality occurs through self-employment activities, although there are countries such as Mexico and Argentina where practically half of those in this group are employees who receive a fixed remuneration.

## Literature review

Informality is widespread and comprises more than two-thirds of employment in emerging market and developing economies [16, 17].

There is an abundance of definitions of informality. The dominant approach in the literature has employed the legalistic definition, according to which informal workers are those without a formal labor contract, and informal firms are those that do not comply with labor regulations including providing employment benefits [18] such as social security, health services, or unemployment insurance. In the same vein, the lack of tax collection from the informal sector can harm the governments’ finances and worsen the provision of public goods.

The literature has shown that informal firms are on average smaller, pay lower wages, are led by individuals with lower levels of education, with less educated workers, and lower profits than their counterparts in the formal sector [19, 20]. As for workers, informal employment is larger among younger and older workers, among women, and decreases with education [16, 19, 20]. The employment category with the highest share of informal workers in developing and emerging economies is own-account and family workers. Worldwide, persons living in rural areas are twice as likely to be in informal employment as those in urban areas, and agriculture is the sector with the highest level of informal employment [16].

There are three main schools of thought about the origins of informality. According to one view, informality is the response to overly burdensome restrictions [21, 22]. Firms are potentially productive, but end up operating in the informal sector because the costs of complying with regulations are too high. Another view sees informality and formality as substantially different. The workers and firms characterized by low productivity are unable to access wage employment or pay the costs of formality from taxes and regulations and hence become informal as a survival strategy, where informality is preferred to unemployment [23–25].

A third view posits that workers and firms who can formalize do so, but those who exhibit lower productivity rationally choose not to because, for them, costs outweigh the benefits of the public services offered in exchange [26–28]. This view has been referred to as the “parasite view”, as these actors are productive enough to survive in the formal sector, but prefer not to, since informality gives them a cost advantage over formal firms. As a result, informal firms take a bigger market share from more productive firms that operate in the formal sector, hindering growth.

The three theories were seen for some time as conflicting but are today viewed as complementary [18, 19, 29], with different firms optimally choosing whether to comply with regulations depending on their characteristics and on the environment that they face, such as laws, regulations, enforcement, welfare policies or the economic cycle [20]. The literature has also focused on the latter aspect by analyzing the dynamics of informality over the business cycle. Informal employment is mostly found to be counter-cyclical, that is, it increases during economic downturns and reduces in economic expansions [7, 19, 20, 30, 31].

## Background and context

In what follows, we discuss the labor market dynamics over the last 15 years for sixteen countries in the Latin American region. The main source of data are the household surveys and the Labor Markets and Social Security Information System (SIMS) database from the Inter-American Development Bank (IDB). Next, using the most recent information available from employment surveys in 2020 in nine countries, we describe the changes in the labor market that occurred during the pandemic. S1 Table lists the surveys used in the analysis.

Hereafter, we define formal employment as that which is registered in institutionalized social security systems financed through a contribution typically divided between employers, workers, and the state (usually called contributory programs). Informal workers are defined as the residual between formal workers and total workers. This definition has been used in Latin America and other regions in multiple studies [19, 32–38].

### Labor market dynamics in Latin America before COVID-19

**Fig 1:**
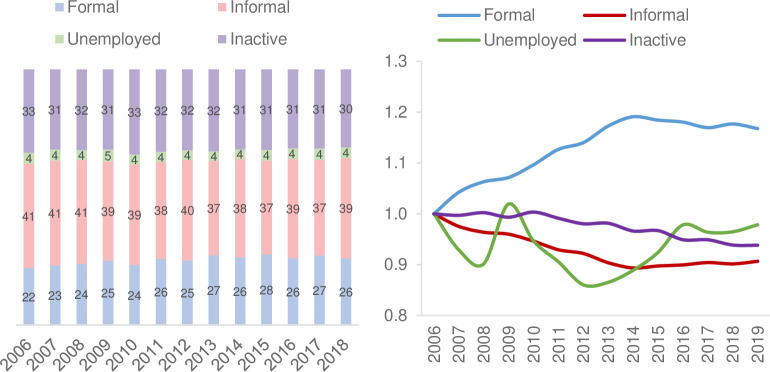
Labor market developments in Latin America. (A) Percentage Distribution of the working-age population. (B) Labor Market Dynamics (2006 = 100). Source: Prepared with data from the IDB’s Labor Markets and Social Security Information System (SIMS) database, 2020. Latin America is a simple average with information from 16 countries.

The percentage of the working-age population in the formal sector in Latin American economies increased consistently between 2006 and 2015, from 22.3 to 27.6 percent of the total (Fig 1A). Correspondingly, the percentage of informal employment decreased from 40.7 to 36.8 percent in those years, while unemployment oscillated around 4 percent. On the other hand, there was a slightly decreasing trend in the percentage of the population classified as "inactive" (neither employed nor seeking to enter the labor market), which is attributed to a long-term underlying trend of increased labor participation of women and youth. These trends came to a halt in 2015. Since then, the percentage of the working-age population in formal and informal jobs has remained stable with marginal variations, the unemployment rate has increased, and the long-term trend decline in the proportion of inactive population has continued (Fig 1B).

**Fig 2:**
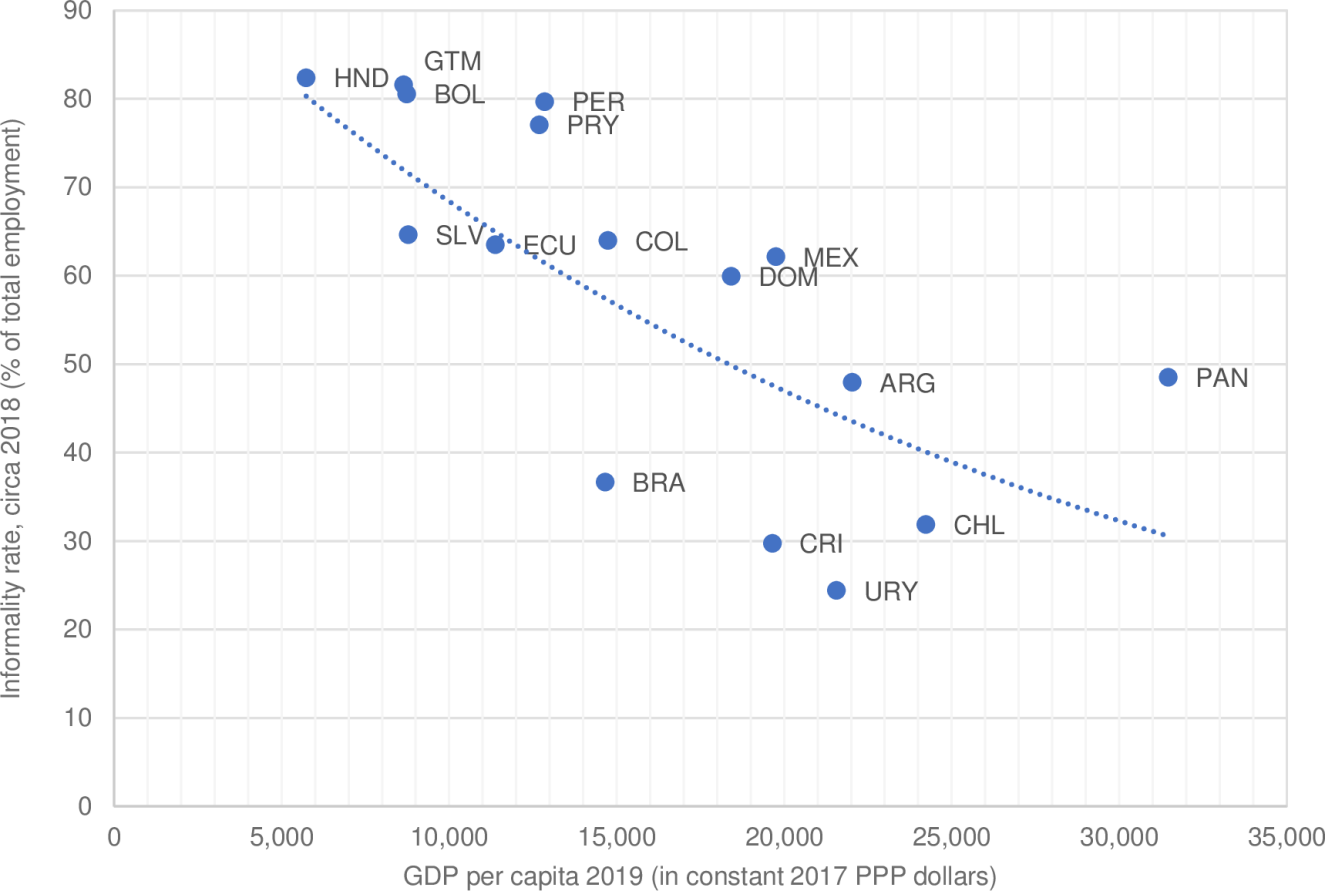
Informality rate of the population age 15 and older and GDP per capita in Latin America. Sources: Based on household survey data and on data from the World Bank’s World Development Indicators. PPP: purchasing power parity. ARG: Argentina, BOL: Bolivia, BRA: Brazil, CHL: Chile, COL: Colombia, CRI: Costa Rica, ECU: Ecuador, SLV: El Salvador, GTM: Guatemala, HND: Honduras, MEX: Mexico, PAN: Panama, PRY: Paraguay, PER: Peru, DOM: Dominican Republic, URY: Uruguay.

Among the countries in the region, the level of informality varies substantially. Fig 2 shows the inverse relationship between GDP per capita and informality rates by country in 2018/2019. Countries above (below) the average ratio line exhibit higher (lower) percentages than expected given their level of development. Of the 16 countries in the sample, 10 have informality rates above 50 percent, with the highest levels in Honduras, Guatemala, and Bolivia – which also have the lowest GDP per capita. In the opposite end Chile, Uruguay and Costa Rica show the lowest informality rates and high GDP per capita.

Within each country, there are disparities across population groups. S2 Table presents the detailed socio-demographic profile of the employed population over 15 years of age around 2018, the population employed in the informal sector, and the population employed in firms with fewer than 5 workers for the 16 countries for which recent household or employment surveys are available. On average, the employment rate for the population over 15 years old is 61.1 percent, ranging from 54.5 in Costa Rica to 69.3 percent in Peru. Concerning employment, in general, women have lower rates than men, with gender gaps above 30 percentage points in Guatemala, Honduras, El Salvador, and Mexico. On the other hand, lower participation rates are observed among the population ages 15 to 24 in Chile, Argentina, Costa Rica, and Uruguay (S2 Table). The percentage of men and women employed in the informal sector is similar on average in Latin America, with more marked differences in Bolivia, Colombia, El Salvador, and Peru. A greater propensity for informality is observed in rural areas and among the population between 15 and 24 years of age.

Differences are also observed in the percentage of people working in companies with 1 to 5 workers, ranging from 83 percent of total employment in Ecuador to 32 percent in Chile. It is noteworthy that the countries with the highest percentages of workers in companies with 1 to 5 workers tend to be the countries with the highest levels of informality (S2 Table). Also, in most countries in the sample, a larger percentage of employed women and people between 15 and 24 years work in enterprises with five or less workers compared to their peers, suggesting that women and youth tend to work in more vulnerable employments.

S3 Table shows the socio-demographic profile of the population over 15 years of age according to inactivity and unemployment. Argentina, Brazil, and Colombia are the countries in the region with the highest unemployment rate. In particular, the cohort aged 15 to 24 years presents a higher unemployment rate–that is, they do not have a job and report that they are looking for work, according to survey information–compared to the other cohorts. In contrast, the highest rates of inactivity are observed among women and the 15–24-year-old cohort. The Central American countries register the highest percentage of inactivity rates among women. This result is consistent with data reported in other studies on the demographic profile of people who neither study nor work in the region [39]. Table 1 summarizes the main changes between 2006 and 2019 in the distribution of the working-age population. Guatemala, Honduras, El Salvador, and Mexico exhibit a pattern different from the average, with reductions in the percentage of the working-age population employed in the formal sector that are reflected in increases in the percentage of informality. Bolivia, Ecuador, Paraguay, Peru, and Colombia display slight decreases in the percentage of the unemployed working-age population, while in Argentina, Chile, Brazil, and Costa Rica the unemployment rate increased. The detailed structure of the working-age population by country for the 2006–2019 period is shown in S4–S7 Tables, which corroborate that there is heterogeneity in the region.

**Table 1:** Absolute change in the distribution of the working-age population in Latin America, 2006–-circa 2018 (percentage points).

Country	Formal	Informal	Unemployed	Inactive
Argentina^a^	2.6	-2.2	0.1	-0.4
Chile	5.2	-1.2	0.8	-4.8
Dominican Republic	12.2	-5.0	1.2	-8.4
Brazil	5.4	-10.2	2.2	2.4
Costa Rica	3.4	-6.0	2.2	0.5
El Salvador	-1.5	2.7	0.3	-1.5
Guatemala	-2.0	4.8	0.1	-2.8
Honduras	-0.6	5.4	0.1	-4.9
Mexico	-1.8	3.9	-0.2	-2.0
Bolivia	4.1	-10.2	-3.5	4.7
Ecuador	8.4	-10.5	-0.2	2.3
Colombia	9.1	-0.9	-2.6	-5.8
Paraguay	8.9	-4.4	-0.6	-4.0
Peru	7.0	-5.6	-0.4	-1.0
Panama	5.3	0.9	-0.6	-5.6
Uruguay	8.2	-6.0	-1.3	-0.9
Average for Latin America	4.6	-2.6	-0.1	-2.0

Source: Prepared with data from the IDB’s Labor Markets and Social Security Information System (SIMS) database, 2020. a The EPH survey for Argentina only has urban coverage.

Several studies that previously analyzed the dynamics of informality in Latin America during the 2000s have shown similar profiles, attributing fluctuations mostly to the economic cycle [40–42]. Other studies analyze the cases of specific countries during the economic cycle, such as Uruguay [43] and Mexico [30, 31]. In this respect, the literature that analyzed the dynamics of informality over the business cycle suggests that informal employment is mostly counter-cyclical, that is, it increases during economic downturns and falls in economic expansions [19, 30, 44, 45]. Also, the literature has found that the Okun coefficient, i.e., the elasticity of unemployment to GDP growth, decreases with the level of labor informality, as informality plays an important role as a shock absorber with the informal-formal margin, limiting movements in the employed-unemployed margin [7, 46, 47]. In other words, informality can act as a “safety net of last resort” [48], or as an escape valve for the labor market in periods of downturns [7, 46, 49]. However, it is also important to bear in mind that although the low elasticity of unemployment to GDP growth can mitigate negative effects of shocks [29], large shares of informality can have other adverse consequences, such as excess output volatility [31] and greater welfare losses [47].

Other research point out that intends to explain some of the observed trends point out that public policies that promote formalization–including those focused on improving productivity, establishing incentives, and strengthening oversight–have played an important role in the observed trends, although results depend on each country [50]. In the 2002–2012 period, on average, 60 percent of the reduction in informality in the region was associated with changes in the economic structure and the remainder with the institutional policies implemented [51].

### Labor market dynamics in 2020

In 2020, nine Latin American countries conducted household and/or employment surveys that allow for examining the evolution of the labor market during the pandemic. During the months of confinement, formal employment grew relative to informal employment. On average, between the first and second quarters of 2020, the ratio of formal to informal employment increased from 0.84 to 1.09, with the most pronounced changes in Argentina, Chile, and Brazil (Table 2). These relative increases in formal employment as a percentage of the working-age population in almost all countries between the first and second quarters of 2020 are a result of the massive exit of informal workers from the labor force.

**Table 2:** Ratio of persons employed in the formal sector relative to the informal sector, selected Latin American countries, 2020.

Country	2019	2020:Q1	2020:Q2	2020:Q3
Argentina^a^	0.9	1.03	1.64	
Brazil	1.8	1.73	1.97	1.87
Chile	2.4	1.66	2.28	2.13
Colombia	0.6	0.61	0.64	0.60
Ecuador	0.7	n.a.	0.45	0.33
Mexico	0.5	0.61	0.73	0.67
Paraguay	0.3	0.30	0.33	0.30
Peru	0.3	0.61	0.65	
Average for Latin America (8 countries)	0.9	0.84	1.09	0.98

Sources: Estimates from household or employment surveys: Argentina—EPH (2019, 2020:Q1, Q2), Bolivia—ECH (2019, 2020:Q1, Q2), Brazil—PNADC (2019, 2020:Q1, Q2, Q3), Chile—ENE (2019, 2020:Q1, Q2, Q3), Colombia—GEIH (2019 and February, June, September 2020), Mexico—ENOE (2019, 2020:Q1, Q3) and ETOE (2019. 2020:Q2), Paraguay—EPHC (2019, 2020:Q1, Q2, Q3), Peru—ENAHO (2019, 2020:Q1, Q2). For Latin America, the data is the simple average for 8 countries with available data. For Peru, the data for the third quarter was not yet published when this paper was written.

^a^ The EPH survey for Argentina only has urban coverage.

**Fig 3:**
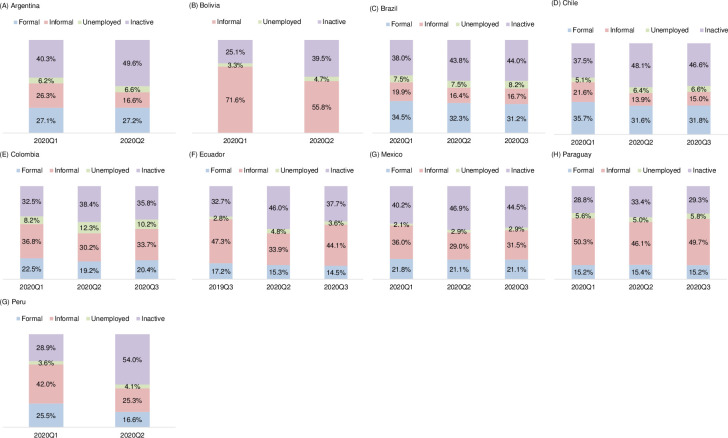
Distribution of the working-age population over 15 Years Old, selected Latin American countries, 2020 (percent). Sources: Estimates from household or employment surveys: Argentina—EPH (2020:Q1, Q2), Bolivia—ECH (2020:Q1, Q2), Brazil—PNADC (2020:Q1, Q2, Q3), Chile—ENE (2020:Q1, Q2, Q3), Colombia—GEIH (February, June, September 2020), Mexico—ENOE (2020:Q1, Q3) and ETOE (2020:Q2), Paraguay—EPHC (2020:Q1, Q2, Q3), Peru—ENAHO (2020:Q1, Q2). The EPH survey for Argentina only has urban coverage. For the second quarter, Bolivia’s ECH survey only reports a limited set of variables, so the formality variable cannot be constructed. For Ecuador, the 2019:Q3 survey is used because 2020:Q1 data are not available.

Fig 3 shows the distribution of the working-age population by country. The majority of the countries in the sample registered an increase in the percentage of the working-age population in inactivity, starting from the second quarter of 2020 –which is the period in which most countries implemented the lockdown measures [52]. Along these lines, data from the Government Response Stringency Index created by the Oxford COVID-19 Government Response Tracker [53] shows that Argentina, Bolivia, Peru, Ecuador, Colombia, and Paraguay were among the countries which implemented a strictest set of response measures to the pandemic.

The results displayed in Fig 3A show that between the first and second quarters of 2020 in urban areas in Argentina, the percentage of the working-age population employed in the formal sector remained relatively constant, but the percentage employed in the informal sector declined by 9 percentage points. The percentage of inactivity increased by 10 percentage points in the same period. For Bolivia, the percentage of the employed population decreased by 16 percentage points in the first two quarters of 2020, with an increase in inactivity and unemployment (Fig 3B). In Brazil, inactivity increased, and the percentage of the employed and unemployed working-age population both in the formal and informal sectors decreased (Fig 3C). For the third quarter of 2020, the data show a slight increase in the percentage of the unemployed and the employed in the informal sector.

In Chile, informality declined by 8 percentage points and the inactive working-age population increased by 10 percentage points between the first and second quarters of 2020 and then stayed high in the third quarter (Fig 3D). In Colombia, the percentage of the working-age population employed in the informal sector decreased by 7 percentage points between the first two quarters of 2020, while formal employment fell by 4 percentage points (Fig 3E). As in the case of other countries, changes in the percentage of the population employed were offset by increases in inactivity and unemployment rates. For the third quarter, Colombia saw a rise in the percentage of the working-age population employed in the informal and formal sectors. Fig 3F provides the data for Ecuador, showing similar labor market dynamics to the rest of the countries in the region, reporting an increase of 13 percentage points in the inactive population, which is mainly explained by the decrease of the population employed in the informal sector.

Mexico shows a similar pattern, with a 7 percentage point reduction in the percentage of the employed working-age population in the informal sector, and an increase of the same magnitude in the percentage of the inactive population between the first two quarters of 2020 (Fig 3G). The third quarter saw an increase in the percentage of informal employment, equivalent to the reduction of the inactive population. Fig 3H shows that in Paraguay, fluctuations in the dynamics of the labor market in the first two quarters of 2020 involved the percentage of the working-age population employed in the informal sector and the inactive population, and it is noteworthy that during the third quarter of 2020, the distribution of the working-age population was similar to the first quarter of the year. Finally, Peru registered the highest increase in inactivity, by 25 percentage points between the first and second quarters of the year (Fig 3I).

These trends are in line with other studies [6] which argue that the changes in the labor market during the pandemic are not reflected in increases in the unemployment rate. Instead, due to mobility restrictions and confinement measures, a large proportion of the employed population left the labor force at least temporarily, increasing inactivity. However, in other regions of the world, the adjustment patterns have been different. In Asian countries, for example, the adjustment seems to be concentrated in increases in informality [54].

The household and employment surveys in all countries also generally show that during the months of confinement in 2020, women, people with fewer years of schooling, and the 15–24 year-old cohort had the highest rate of transition from informality to inactivity (Table 3). Particularly for the latter group, fluctuations in labor dynamics may have long-term consequences, since according to the empirical evidence available for Latin America, cohorts that enter the labor market in periods of recession are more likely to work in jobs with lower incomes and have less access to social security in the long run [55–58].

**Table 3:** Demographic profile of the population employed in the informal sector as a percentage of employment, selected Latin American countries, 2020 (percent).

Country	Total	Women	Men	Urban	Rural	15–24	25–49	50–64	No Schooling	Primary	Lower Secondary	Upper Secondary	Tertiary Education
2020:Q1													
Argentina (urban)^a^	49.3	49.0	49.4			68.6	44.0	44.0	92.3	68.4	66.7	49.0	34.5
Bolivia^b^	87.1	88.3	86.1	82.6	95.8	95.6	82.6	87.7	96.8	97.8	92.3	82.0	70.4
Brazil	36.6	35.2	37.7	36.6	36.4	46.4	32.5	37.1	74.3	58.5	49.6	34.1	19.2
Chile	37.7	38.7	36.9	36.1	49.6	43.6	32.5	40.9	49.2	58.6	50.4	35.5	30.2
Colombia	62.0	60.5	63.0	56.6	81.7	71.7	71.7	67.3	94.7	84.6	80.8	61.0	32.2
Mexico	62.3	61.4	62.8	51.5	72.1	68.1	56.1	67.6	93.7	90.5	72.8	61.0	44.4
Paraguay	76.7	74.2	78.6	70.5	88.0	83.9	70.8	84.4	98.5	92.5	87.3	76.2	52.3
Peru	62.2	70.5	55.6	54.9	90.7	80.2	56.1	61.6	96.9	87.9	82.9	68.0	36.9
2020:Q2													
Argentina (urban)a	37.8	36.7	38.7			59.8	34.1	36.8	25.5	53.3	58.5	39.5	24.7
Brazil	33.7	31.6	35.3	33.8	33.2	44.7	29.7	33.9	71.2	56.7	46.6	32.2	17.3
Chile	30.5	29.8	31.1	29.1	42.7	35.8	26.7	33.8	49.7	48.4	44.9	28.5	25.2
Colombia	61.2	57.5	63.5	53.6	86.5	70.7	54.4	66.3	94.5	87.2	84.2	60.4	29.5
Ecuador^c^	69.0	68.2	69.5	59.4	85.8	81.2	62.8	71.5					
Mexico	57.9	55.0	59.8	44.4	68.2	61.9	51.8	63.2	84.7	87.0	69.6	54.6	40.2
Paraguay	74.9	74.5	75.2	67.9	87.0	83.2	69.4	79.2	96.0	93.1	87.4	75.0	48.8
Peru	60.4	67.3	55.8	48.1	88.7	83.1	54.7	58.1	96.1	87.7	81.2	66.4	27.1
2020:Q3													
Brazil	34.8	32.2	36.9	34.4	36.7	46.7	31.0	34.8	72.8	57.4	49.4	33.8	17.5
Chile	32.0	31.8	32.1	30.8	42.1	37.5	28.2	34.7	71.6	50.8	44.0	31.2	25.6
Colombia	62.4	59.8	64.0	56.0	85.6	73.5	55.4	66.5	91.8	86.6	83.0	62.4	31.9
Ecuador^c^	75.3	75.2	75.3	68.0	88.8	86.9	67.3	78.7	96.4	90.3	87.3	85.8	46.6
Mexico	59.9	56.9	61.7	47.9	70.5	68.5	53.8	63.9	94.1	90.6	71.5	62.7	41.7
Paraguay	76.6	74.5	78.0	70.0	87.6	84.5	70.6	83.1	99.1	94.0	86.1	78.2	51.0

Sources: Estimates from household or employment surveys: Argentina—EPH (2020:Q1, Q2), Bolivia—ECH (2020:Q1, Q2), Brazil—PNADC (2020:Q1, Q2, Q3), Chile—ENE (2020:Q1, Q2, Q3), Colombia—GEIH (February, June, September 2020), Ecuador–ENEMDU (2020:Q2, Q3), Mexico—ENOE (2020:Q1, Q3) and ETOE (2020:Q2), Paraguay—EPHC (2020:Q1, Q2, Q3), Peru—ENAHO (2020:Q1, Q2).

^a^ The EPH survey for Argentina only has urban coverage.

^b^ For the second quarter, Bolivia’s ECH survey only reports a limited set of variables, so the formality variable cannot be constructed.

^c^ Data are not available for the first quarter of the ENEMDU survey for Ecuador. The May/June 2020 telephone survey contains only a limited number of variables.

Analyzing the results displayed in Table 3 by country, in Argentina, the percentage of women employed in the informal sector decreased more sharply than the men employed in the informal sector between the first and second quarter of 2020. However, in the third quarter, the share of men working in the informal sector increased more rapidly than women. Chile and Mexico show a similar downward trend for the women employed in the informal sector during the second quarter and a slight increase in the third quarter. In Brazil, Colombia, and Peru, the share of women employed in the informal sector decreased around 3 percentage points in the three countries. In all countries with available information, the share of the urban population working in the informal sector declined in the second quarter compared to the rural areas–Chile, Mexico, and Peru are the countries with the highest decrease of informal employment in the urban areas. By age cohort, between the first and second quarter of 2020, the percentage of the population of 15 to 24 years old employed in the informal sector decreased by 8.8, 7.8, and 6.2 in Argentina, Chile, and Mexico, respectively. In Brazil and Paraguay, the share of the population ages 50 to 64 employed in the informal sector was the cohort that registered the sharpest decrease in employment compared to the other age cohorts. The results for educational attainment show that a larger share of workers with primary education or less are mainly employed in the informal sector.

Table 4 presents the profile of the unemployed population during 2020. Men and those in the ages of 15–24 age group constituted a higher percentage of the working-age population in this status, and their numbers increased between the first two quarters of the year. In Bolivia, Chile, and Mexico, the share of unemployed men increased in the second quarter of 2020 by more than 2 percentage points, but the largest rise was registered in Colombia with 5 percentage points during the same period. In Bolivia, the urban unemployment rate decreased by 4.3 percentage points in the second quarter. The age cohort of 24 to 49 years old showed the highest unemployment rate increase in the second quarter in Colombia, followed by Chile and Peru. By educational attainment, the population with upper-secondary or less registered the highest unemployment rate increase in Colombia, whereas in Bolivia and Paraguay this group showed a downward trend.

**Table 4:** Demographic profile of the unemployed population as a percentage of the working-age population, selected Latin American countries, 2020.

Country	Total	Women	Men	Urban	Rural	15–24	25–49	50–64	No Schooling	Primary	Lower Secondary	Upper Secondary	Tertiary Education
2020:Q1													
Argentina (urban)^a^	6.2	5.6	6.8			10.6	6.8	4.9	2.4	4.4	6.9	8.2	5.0
Bolivia^b^	3.3	3.1	3.5	4.3	1.0	4.8	3.9	1.6	1.6	1.4	3.4	3.8	5.2
Brazil	7.5	7.7	7.4	7.5	7.5	16.0	8.1	3.8	2.2	4.4	7.9	10.6	6.5
Chile	5.1	5.1	5.2	5.4	3.3	7.0	6.5	3.8	1.7	1.7	4.7	5.7	6.6
Colombia	8.2	9.2	7.2	9.1	4.9	11.5	9.6	5.2	2.4	4.3	7.4	10.3	10.9
Mexico	2.1	1.5	2.6	2.6	1.6	3.0	2.5	1.0	0.4	0.8	1.7	2.4	3.0
Paraguay	5.6	6.0	5.3	6.3	4.5	10.3	5.2	2.4	0.2	3.4	6.5	8.1	5.7
Peru	3.4	3.5	3.4	4.0	0.5	5.6	3.4	2.2	0.3	0.6	2.4	2.8	5.9
2020:Q2													
Argentina (urban)^a^	6.6	5.7	7.6			8.9	8.2	4.8	1.7	6.5	6.8	7.4	5.7
Bolivia	4.7	3.8	5.7			4.7	6.0	2.9					
Brazil	7.5	7.0	8.0	7.5	7.3	14.6	8.6	4.1	2.5	4.6	7.8	10.4	6.3
Chile	6.4	4.8	7.9	6.5	4.9	6.2	9.0	4.9	1.2	2.6	5.4	7.1	7.5
Colombia	12.3	12.3	12.2	14.0	5.6	14.3	15.2	9.1	2.9	7.1	12.3	15.3	14.7
Ecuador^c^	4.8	4.1	5.6	6.1	1.9	5.5	6.8	3.1					
Mexico	2.9	1.9	4.0	3.4	2.5	3.6	3.6	2.0	1.6	0.8	3.3	2.2	3.3
Paraguay	5.0	4.6	5.5	6.0	3.3	8.7	5.0	2.1	1.7	2.9	5.2	7.4	5.3
Peru	4.0	2.7	5.4	4.7	0.7	4.0	5.5	2.4	1.0	1.0	2.1	4.3	6.3
2020:Q3													
Brazil	8.2	7.8	8.6	8.2	8.2	15.7	9.7	4.5	2.3	4.9	8.3	11.6	6.8
Chile	6.6	5.0	8.3	6.8	4.7	6.0	9.3	5.2	0.3	2.5	5.3	7.7	7.4
Colombia	10.2	10.8	9.5	11.6	4.8	12.2	12.7	7.3	2.5	4.9	9.1	13.1	13.2
Ecuador^c^	3.6	3.6	3.6	4.5	1.5	4.3	5.1	2.2	0.6	1.3	3.3	4.9	5.7
Mexico	2.9	1.9	3.9	3.5	2.2	3.7	3.5	2.0	0.9	1.4	2.4	2.9	3.8
Paraguay	5.8	6.6	4.9	7.1	3.4	9.9	5.6	2.6	1.7	2.6	5.9	9.1	6.3

Sources: Estimates from household or employment surveys: Argentina—EPH (2020:Q1, Q2), Bolivia—ECH (2020:Q1, Q2), Brazil—PNADC (2020Q1, Q2, Q3), Chile—ENE (2020:Q1, Q2, Q3), Colombia—GEIH (February, June, September 2020), Ecuador–ENEMDU (2020:Q2, Q3), Mexico—ENOE (2020:Q1, Q3) and ETOE (2020:Q2), Paraguay—EPHC (2020:Q1, Q2, Q3), Peru—ENAHO (2020:Q1, Q2).

^a^ The EPH survey for Argentina only has urban coverage.

^b^ For the second quarter, Bolivia’s ECH survey only reports a limited set of variables, so the formality variable cannot be constructed.

^c^ Data are not available for the first quarter of the ENEMDU survey for Ecuador. The May/June 2020 telephone survey contains only a limited number of variables.

In the first two quarters of 2020, there was a larger increase in the inactivity rate of young people (Table 5). This result, combined with what was discussed above, suggests that more young people exited the labor force. The data disaggregated by sex reveal that in Colombia, Paraguay, and Peru, the percentage of women in a condition of inactivity increased in greater proportion than men in the same period. In Peru, Chile, and Mexico, the share of the inactive population in urban areas increased more than in rural areas between the first and second quarters of 2020. Consistent with the previous discussion, the population between 15 to 24 years old displayed the largest rise in the inactive share of the population, particularly in Peru, Bolivia, and Chile, with the values remaining above the pre-pandemic levels for the majority of the countries during the third quarter.

**Table 5:** Demographic profile of the inactive population as a percentage of the working-age population, 2020.

Country	Total	Women	Men	Urban	Rural	15–24	25–49	50–64	No schooling	Primary	Lower Secondary	Upper Secondary	Tertiary education
2020:Q1													
Argentina (urban)^a^	40.3	49.6	29.8			61.3	17.8	30.6	75.0	51.7	49.7	38.2	28.0
Bolivia^b^	25.1	32.3	17.5	29.5	14.1	43.7	13.3	15.9	31.0	19.9	23.8	34.1	22.1
Brazil	38.0	47.1	27.9	38.1	38.0	44.9	19.0	41.1	77.0	55.2	43.4	30.0	21.6
Chile	37.5	47.9	26.7	37.0	41.5	64.4	18.8	29.3	62.5	61.9	43.6	33.0	27.4
Colombia	32.5	44.5	19.6	31.8	35.1	49.7	15.6	29.0	53.1	39.5	42.2	28.6	20.3
Mexico	40.2	55.1	23.6	39.2	41.1	56.3	24.4	37.8	62.4	50.7	41.1	54.8	30.4
Paraguay	28.8	40.9	16.2	28.1	30.1	42.8	15.3	26.1	61.7	33.1	36.9	27.3	14.7
Peru	27.9	35.8	20.0	29.8	18.1	45.2	17.1	19.6	35.5	26.2	31.2	31.8	23.9
2020:Q2													
Argentina (urban)^a^	49.6	58.0	40.4			73.9	28.7	42.1	83.1	61.3	60.5	49.7	33.7
Bolivia	39.5	48.0	30.4			66.6	23.3	28.2					
Brazil	43.8	53.0	33.4	43.8	44.0	53.1	24.9	46.0	80.8	60.5	51.2	37.0	26.7
Chile	48.1	58.8	36.9	47.4	53.4	78.0	28.9	40.2	88.4	72.3	56.3	44.6	35.8
Colombia	38.4	50.6	25.4	37.9	40.2	55.4	21.0	36.8	58.7	46.7	48.9	34.6	24.9
Ecuador^c^	46.0	58.0	33.5	49.0	39.2	66.5	28.0	38.7					
Mexico	46.9	60.3	32.0	49.8	44.5	64.2	31.2	44.2	65.4	56.5	48.0	64.4	36.9
Paraguay	33.4	47.5	18.8	33.4	33.4	48.7	19.8	29.7	59.0	37.6	39.7	34.4	20.0
Peru	53.4	62.6	44.1	59.4	24.7	69.9	43.6	46.4	55.2	45.2	58.8	58.2	51.7
2020:Q3													
Brazil	44.0	53.5	33.1	43.8	44.6	52.1	24.1	46.7	81.1	60.9	52.1	36.4	26.9
Chile	46.6	57.3	35.5	45.8	53.0	77.9	27.1	38.2	88.3	72.7	57.7	42.0	35.4
Colombia	35.8	47.9	22.9	34.7	39.7	50.8	18.5	32.3	58.2	43.9	48.3	30.0	23.7
Ecuador^c^	37.7	48.8	26.2	40.5	31.3	58.5	19.2	27.6	52.7	37.0	32.4	54.2	31.4
Mexico	44.5	60.1	27.5	44.7	44.3	59.3	29.1	43.6	66.0	55.1	45.9	57.5	35.6
Paraguay	29.3	42.5	15.7	30.2	27.7	44.0	16.2	25.2	57.2	33.8	37.8	28.7	16.4

Sources: Estimates from household or employment surveys: Argentina—EPH (2020:Q1, Q2), Bolivia—ECH (2020:Q1, Q2), Brazil—PNADC (2020Q1, Q2, Q3), Chile—ENE (2020:Q1, Q2, Q3), Colombia—GEIH (February, June, September 2020), Ecuador–ENEMDU (2020:Q2, Q3), Mexico—ENOE (2020:Q1, Q3) and ETOE (2020:Q2), Paraguay—EPHC (2020:Q1, Q2, Q3), Peru—ENAHO (2020:Q1, Q2).

^a^ The EPH survey for Argentina only has urban coverage.

^b^ For the second quarter, Bolivia’s ECH survey only reports a limited set of variables, so the formality variable cannot be constructed.

In sum, preliminary results for 2020 suggest that, in the context of the health and economic crisis, a greater proportion of the economically active population left the labor market and became inactive, with most of the jobs lost corresponding to the informal sector. According to the survey calculations, in Argentina, Colombia, Mexico, and Paraguay there was a greater outflow of dependent employees, but with informal employment contracts, while in Brazil, Chile, Ecuador, and Peru, the drop among the self-employed was greater than that observed in this group. The formal sector functioned as a social safety net during the pandemic. This may be due to several reasons, including (i) labor regulations that impose firing costs, (ii) uncertainty about the duration and depth of the health crisis that complicated estimating the costs and benefits of maintaining jobs, (iii) employment support measures introduced by different governments, and (iv) the preference of some employers to reduce the hours of activity per job instead of reducing the production plan, and thus avoiding firing costs.

## Potential effects on the labor market in the future

To explore the possible future dynamics of labor markets in Latin America, this section presents two approaches:

- In the first, a panel of country/years is constructed with aggregate data to measure the elasticity between average wages in each country/year and the corresponding informality rate (macroeconomic approach).
- The second approach estimates the probability of being informal as a function of income and other observable characteristics of individuals and households, based on microdata from the most recent household or employment surveys in each country (microeconomic approach).

Both approaches take as a basis the inverse relationship observed before 2020 between informality and income. Both also start from the consensus that informality in Latin America historically behaved countercyclically [46, 59], and that once the economic recovery begins, that relationship will resume. In this sense, the COVID-19 episode is a one-time anomaly, meaning that before and after its occurrence, long-term patterns will once again take hold. It is also possible to argue the inverse directionality in that formalization can lead to higher wages if access to formalization influences productivity, for example, by opening up the possibility of acquiring credit or insurance, by providing greater stability that can broaden investment horizons, or by granting access to more dynamic and productive markets that operate under legal regulations. As a result, the higher the wages, the lower the informality, and vice versa.

The estimates take as a starting point that changes in wages determine informality. The logic of the wage-informality directionality is based on the notion that when wages are low (reflecting low productivity), it is unfeasible to cover the costs of social security benefits or to pay the minimum wage established by law, so employment takes place without access to such benefits or below the legal minimum. Other empirical studies used a similar argument using the value of the minimum wage as a reference instead of the value of social security contributions [7, 26, 49, 50, 60, 61].

### Panel data estimation

The first approach, with an aggregate macroeconomic focus, is based on the construction of a panel of data for the 16 countries analyzed with information for the period 1990–2019. This panel is used to estimate how changes in economic activity–measured through real wages–are associated with changes in the informality rate of the employed population in Latin America. Specifically, the following fixed effects model is estimated:

$$\begin{array}{l} Ln(Informality_{i,t})=\alpha +\beta_{1}Ln{\left(Wage\right)}_{i,t}+\beta_{2}Ln\left(EAPagriculture_{i,t}\right)+\beta_{3}Ln\left(EAP\;15-24_{i,t}\right)\\ \;+\beta_{4}Edu_{i,t}+\beta_{5}Ln\left(EAP\;women_{i,t}\right)+\delta_{t}+v_{i}+u_{i,t} \end{array}$$ (1)

In Eq (1), the dependent variable, *Ln*(*Informality*_*i*,*t*_0 represents the natural logarithm of the informality rate of the employed population for country *i* in year *t*; *Ln*(*EAPagriculture*_*i*,*t*_) indicates the natural logarithm of the percentage of the economically active population working in the agricultural sector for country *i* in year *t*; *Ln*(*EAP* 15 – 24_*i*,*t*_) is the natural logarithm of the percentage of the economically active population between 15 and 24 years of age for country *i* in year *t*; *Ln*(*EAP women*_*i*,*t*_) is the natural logarithm of the percentage of women in the economically active population for country *i* in year *t*; and *Edu*_*i*,*t*_ corresponds to the average years of education of the working-age population. Finally, *Ln*(*Wage*)_*i*,*t*_ represents the natural logarithm of the average real monthly wage in constant purchasing power parity (PPP) international dollars.

In estimating Eq (1), it is worth noting that endogeneity issues might arise. On the one hand, there is potential simultaneity among the independent variables associated with reverse causality, which could result in the endogeneity of some regressors such as the wages and the percentage of the women in the labor force–variables that might be highly correlated with the informal employment. On the other hand, there is also potential endogeneity because of omitted variables. Although the time and country-specific fixed effects capture the unobserved variables, they could also be correlated with the independent variables. Given this, it should be noted that the model presented above is not exhaustive, and its purpose is not to establish a causal relationship, but rather to illustrate the direction and degree of correlation between the variables. In addition to the baseline regression, we also report the results, the ordinary least squares (OLS) estimator, and a random fixed effects model (RE) including all the explanatory variables.

Column (1) in Table 6 shows the results of the Eq (1) using time and country fixed effects. The results suggests a negative and statistically significant association between the average wage and the informality rate, with an elasticity of -0.2 (a 1 percent increase in average wages is associated with a 0.2 percent decrease in the informality rate). The coefficient of the percentage of the economically active population aged 15 to 24 suggests a positive and statistically significant correlation with the informality rate, consistent with the demographic profile of the employed population discussed earlier in the paper. The rest of the coefficients are not statistically significant. The rest of the columns in Table 6 display the result for the other estimators, in which the coefficient of the average wage remains with the negative sign and is statistically significant.

**Table 6:** Estimate for Latin America of the correlation between the informality rate and the average monthly salary.

Variables	(1)	(2)	(3)
	FE	RE	OLS
Ln (average real monthly salary)	-0.200**	-1.078***	-1.062***
	(0.087)	(0.201)	(0.095)
Ln (percentage of agricultural EAP)	-0.242	-0.059	-0.062***
	(0.153)	(0.061)	(0.018)
Ln (percentage of the EAP aged 15 to 24 years)	0.466*	-0.019	-0.062
	(0.239)	(0.475)	(0.148)
Ln (percentage of women in the EAP)	0.015	-0.004	0.074
	(0.355)	(0.453)	(0.152)
Average years of education of the labor force	-0.006	-0.003	-0.010
	(0.039)	(0.027)	(0.019)
Constant	0.726	6.096***	6.400***
	(0.719)	(1.306)	(0.504)
Time fixed effects	Yes	Yes	No
Country fixed effects	Yes	Yes	No
Observations	316	316	316
R-squared	0.566	0.5571	0.538
Number of clusters	16	16	

Sources: Estimates based on data from the World Bank’s World Development Indicators and the IDB’s Labor Markets and Social Security Information System (SIMS) database, 2020. L1: is the lag of the dependent variable. EAP: economically active population. Robust standard errors in parentheses. ***p<0.01,

**p<0.05,

*p<0.1.

Based on the results of the fixed effects model in column (1) in Table 6, the path of the informality rate is simulated assuming a fall in labor income in 2020 using the relationship between reductions in GDP and changes in wage income in some previous crisis episodes. The International Monetary Fund’s (IMF) GDP projections for the period 2020–2023 are used to forecast how average wage incomes in the region will behave in the years ahead [4]. Finally, the coefficient (elasticity) of real wages of 0.2 is used to simulate how changes in wages will correspond to changes in the informality rate on average for the 16 Latin American countries included in the panel.

**Fig 4:**
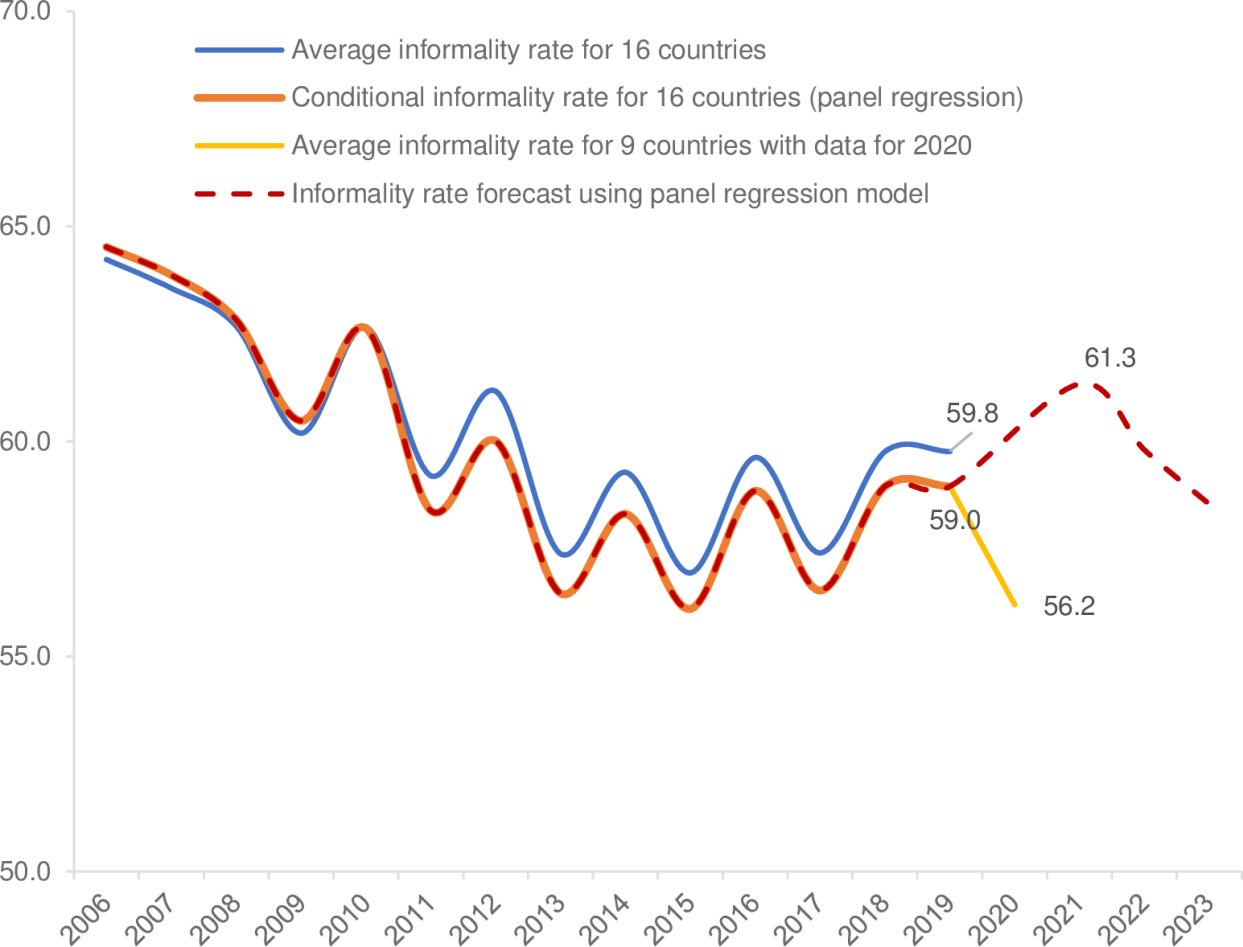
Latin America: Simulation of the informality rate (percent). Source: Estimates based on data from the World Bank’s World Development Indicators; the IDB’s Labor Markets and Social Security Information System (SIMS), 2020; and 2020 employment and household surveys for Argentina, Bolivia, Brazil, Chile, Colombia, Ecuador, Mexico, Paraguay, and Peru. The blue line represents the average informality rate in 16 countries (Argentina, Bolivia, Brazil, Chile, Colombia, Costa Rica, Dominican Republic, Ecuador, El Salvador, Guatemala, Honduras, Mexico, Panama, Paraguay, Peru, Uruguay; the red line is the informality rate predicted by the model in Eq (1); and the yellow line presents the drop in the informality rate between 2019 and 2020 in Argentina, Bolivia, Brazil, Chile, Colombia, Ecuador, Mexico, Paraguay and Peru.

Fig 4 presents the results of this exercise for Latin America. Based on the assumptions described above, the forecast is for the rate of informality to increase in 2021 by 4 percent on average as a result of the fall in wages. This is equivalent to an increase of 2.3 percentage points with respect to the latest regional average in 2019 (from 59 to 61.3 percent).

For a better understanding of the result displayed in Fig 4, we use a first-order Taylor approximation for decomposing the employment [31], yielding the following linear regression:

$$\text{ln}\left(ir_{i,t}\right)=\alpha +\gamma_{1}\;\text{ln}\left(ur_{i,t}\right)+\gamma_{2}\;\text{ln}\left(inactive_{i,t}\right)+\gamma_{3}\;\text{ln}\left(fr_{i,t}\right)+\delta_{t}+v_{i}+u_{i,t}$$ (2)

In Eq (2), ir_it_ is the informal employment as a share of the working-age population, ur_it_ is the unemployment rate, inactive_it_ stands for the inactive share of the working-age population, and fr_it_ is the formal employment as a share of the working-age population (a concise version of Eq (2)). First, Column (1) in Table 7 presents the correlation between employment as a share of the working-age population, the unemployment rate, and the inactive percentage of the working-age population. The results suggest that the employment rate has a larger association with the inactivity rate than the unemployment rate. Column (2) in Table 7 displays the results of Eq (2), where the dependent variable is now the informality rate. The coefficients–which can be interpreted as elasticities—show that informal employment has a larger elasticity to the inactivity rate than to the formal employment since the coefficient for the inactivity rate is 20 percent larger than the formal employment coefficient. This result suggests that informal employment is more sensitive to changes in the inactivity rate. In contrast, the smaller coefficient for the unemployment rate suggests that informal employment in Latin America might be less sensitive to the unemployment rate.

**Table 7:** Estimate for Latin America of the correlation between the informality rate and economic activity status.

Variables	(1)	(2)
	Ln(Employment)	Ln(Informal employment)
Ln(unemployment)	-0.074***	-0.122***
	(0.008)	(0.036)
Ln(inactive)	-0.462***	-0.681***
	(0.023)	(0.187)
Ln(formal)		-0.560***
		(0.102)
Constant	-1.200***	-3.026***
	(0.030)	(0.296)
Observations	393	337
R-squared	0.949	0.634
Number of clusters	16	16
Time fixed effects	Yes	Yes
Country fixed effects	Yes	Yes

Sources: Estimates based on data from the World Bank’s World Development Indicators and the IDB’s Labor Markets and Social Security Information System (SIMS) database, 2020. Robust standard errors in parentheses. ***p<0.01,

**p<0.05,

*p<0.1.

Thus, one possible interpretation of the estimated increase in informal employment reported in Fig 4 –and assuming that the health emergency might be under control—is that most of those who became inactive during COVID-19 will try to re-enter the labor market since informal employment seems to be more sensitive to changes in the inactivity rate (Table 7). In a context of reduced economic growth, the supply of formal jobs will be much more limited, leaving informal activities as the only alternative. A second factor might be related to the fall in average household income, which could push household members who were not active before COVID-19 to seek employment options to restore the pre-COVID-19 level of household income. This would lead to a "wave" of larger labor market entry that would overcompensate for previous employment levels until real wages return to their pre-COVID-19 level. It is therefore foreseen that, in a scenario of rigidity of formal jobs, informality might be the labor market’s "escape valve" to accommodate higher labor supply. The overcompensation effect is most clearly observed when comparing the level of informality recorded in the nine countries with data for 2020 (yellow line in Fig 4), when informality declined by almost 3 percentage points with respect to the previous year. If the level of 56.2 percent observed in these countries in 2020 is taken as a starting point, the growth in informality is almost 3 percentage points higher than the pre-COVID-19 level in 2019. In other words, it is estimated that, once the recovery begins, informality will not return to previous levels immediately, but that there will be an overreaction in labor markets towards greater informality before converging in at least three years to the situation observed before 2020, due to the gradual recovery of wages. The speed with which this increase is reached will depend on the speed with which confinement and social distancing measures are eliminated in each country, allowing economic activity to resume.

In this respect, recent studies on the impact of COVID-19 on labor markets in Latin America seem to be confirming our projections, as shown for Colombia [14] and by the latest report by ILO for Argentina, Brazil, Costa Rica, Chile, Mexico, Paraguay and Peru [6].

### Survey data estimation

In the second approach we use data from household and/or employment surveys (S1 Table) to estimate probability models for each country. In this cross-sectional microeconomic approach, the dependent variable is a binary variable that takes the value of 1 if the individual is employed in the informal sector and 0 otherwise, and the independent variables include labor income and a series of socio-demographic characteristics of the population as control variables. Taking into account that the dependent variable is binary, a probit regression model is used. Estimates are made with robust standard errors and the corresponding expansion factor for each survey.

The specification of the models is as follows:

$$\text{Pr}{\left(Informal=1\;|\;X\right)}_{i}=\Phi (\alpha +\beta_{1}Ln{\left(Income\right)}_{i}+\beta_{2}Sex_{i}+\beta_{3}Rural_{i}+\sum_{i=2}^{5}\gamma_{i}Cohort_{i}+\beta_{5}Edu_{i}+\sum_{i=2}^{9}\delta_{i}Sector_{i}+u_{i})$$ (3)

The variable *Ln*(*income*)_*i*_ represents the natural logarithm of the monthly monetary labor income of the individual’s main occupation. The variable *Sex*_*i*_ takes the value of 1 if the person is male and 0 if the person is female, and *Rural*_*i*_ takes the value of 1 if the person lives in a rural area, and 0 otherwise. The categorical variables of *Cohort*_*i*_ correspond to the 15–24, 25–49, 50–64, and 65 and over age groups, with the 15–24-year-old variable as the base category. The variable *Edu*_*i*_ refers to the person’s years of education. Finally, *Sector*_*i*_ is a dichotomous variable for the nine branches of activity, taking a value of 1 for the occupation branch, and 0 otherwise, with agriculture, forestry, and fishing as the base category.

S8 and S9 Tables present the results of the estimation of the probit model and the marginal effects, respectively, for the 16 countries in the sample. In general, in all countries the marginal effect of the average labor income variable has a negative sign and is statistically significant, suggesting that the higher the income, the lower the likelihood of working in the informal sector. Likewise, for all countries the marginal effect of the years of education variable is negative and statistically significant, suggesting that the higher the education the lower the propensity to work in the informal sector. The rest of the coefficients vary in sign and significance among countries, reflecting the heterogeneity in the profile of the informal population in the region.

**Fig 5:**
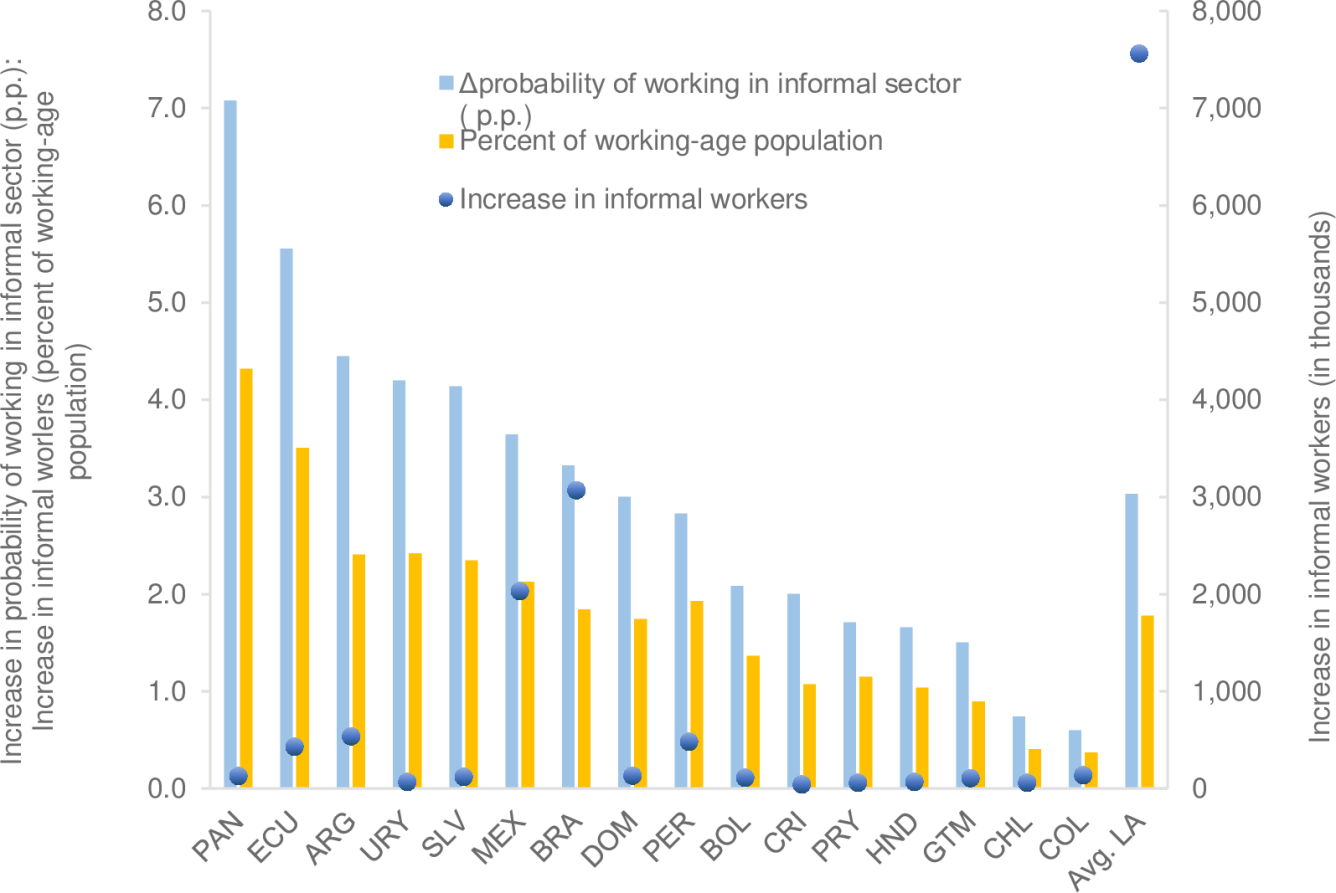
Latin America: Changes in the informality rate associated with declines in labor income compared to the pre-COVID-19 level. Sources: Estimates from household or employment surveys: Argentina—EPH (2019), Bolivia—ECH (2018), Brazil—PNADC (2018), Chile—CASEN (2017), Colombia—GEIH (2018), Costa Rica—ENAHO (2018), Ecuador—ENEMDU (2018), El Salvador—EHPM (2019), Guatemala—ENEI (2018), Honduras—EPHPM—(2018), Mexico—ENIGH (2018), Panama—EPM (2017), Paraguay—EPHC (2018), Peru—ENAHO (2018), Dominican Republic—ENCFT (2017), Uruguay—ECH (2019). The EPH survey in Argentina only has urban coverage. The blue bar indicates the increase in percentage points of the probability of working in the formal sector associated with declines in labor income due to the pandemic (left axis); the yellow bar indicates this increase of informal workers as a percentage of the working-age population (left axis); the green circle indicates the increase of informal workers in absolute numbers (right axis). p.p.: percentage points.

The results of the probit model estimation (S8 Table) are used to simulate various scenarios. A first calculation consists of approximating the effect of the economic crisis following COVID-19 by assuming an adjustment factor for individual labor income using the same GDP/wage ratio, described previously, with which the effect of the decrease in labor income on the informality rate is subsequently simulated. S10 Table displays the parameters used in the simulations. Fig 5 presents the increase in the informality rate associated with declines in labor income due to the pandemic in each country. On average, in Latin America, the result suggests an increase of 3 percentage points in the informality rate with respect to the pre-pandemic level–equivalent to almost 7.6 million people–with values ranging from 0.6 percentage points for Colombia to 7 percentage points for Panama. It is interesting to note that the predicted change is similar to that estimated under the panel data estimation of Fig 4, which is 2.3 percentage points.

This exercise also illustrates how changes in sectoral composition could be associated with changes in the informality rate. In this regard, if during the economic recovery a larger proportion of the population were to be employed in less productive sectors and more likely to be informal, one would expect an increase in the informality rate in addition to that estimated by the change in the average wage.

To explore this aspect in greater detail, a simulation is carried out based on the survey data regarding the composition of the employed population in each country by formality status (with pre-pandemic data) and branch of economic activity (with 2020 surveys). In general, all countries show a higher percentage of the formal population employed in social and communal services activities–which include public administration, teaching, and social and health services, among others–while the trade, restaurants and hotels, and agriculture and livestock sectors have the highest proportion of informal employment. For the five countries in the region with data available for 2020 by economic activity, the percentage of the population working in commerce and services in the informal sector decreases slightly between the first and second quarters of 2020.

**Fig 6:**
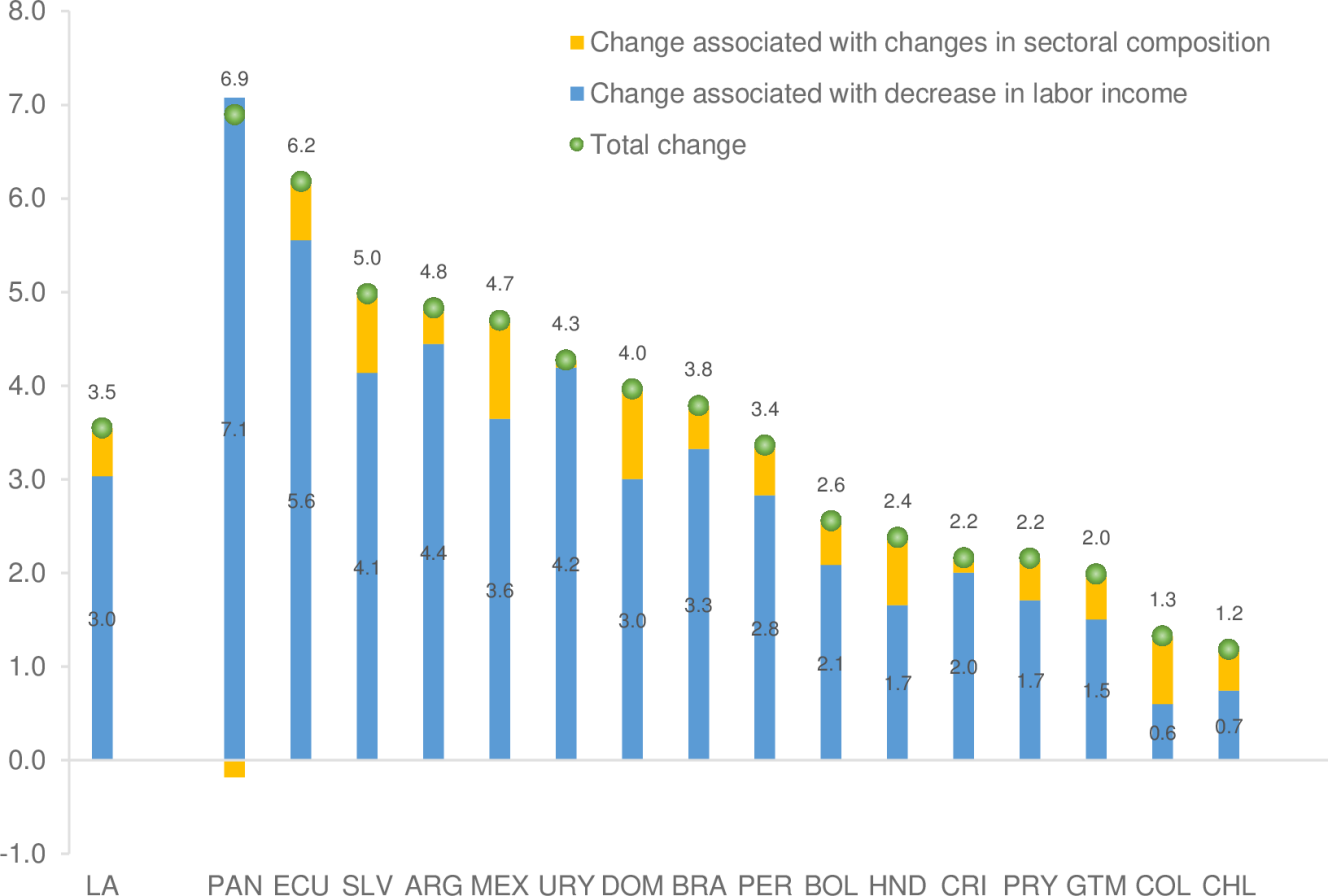
Latin America: Simulation of changes in the informality rate associated with changes in labor income and in the sectoral composition of the employed population (percentage points). Source: Estimates from household or employment surveys: Argentina—EPH (2019), Bolivia—ECH (2018), Brazil—PNADC (2018), Chile—CASEN (2017), Colombia—GEIH (2018), Costa Rica—ENAHO (2018), Ecuador—ENEMDU (2018), El Salvador—EHPM (2019), Guatemala—ENEI (2018), Honduras—EPHPM—(2018), Mexico—ENIGH (2018), Panama—EPM (2017), Paraguay—EPHC (2018), Peru—ENAHO (2018), Dominican Republic ENCFT (2017), Uruguay—ECH (2019). The EPH survey in Argentina only has urban coverage.

Using this information, it can be shown for illustrative purposes what would happen to formality and informality rates if there were an increase of 5 percentage points in the share of the population employed in commerce and hotel and restaurant activities, and a decrease of 5 percentage points in the share employed in manufacturing, which is what generally occurred during previous GDP contractions. Under these circumstances, the average increase in the informality rate would be 3.5 percentage points, of which 0.5 percentage points corresponds to the change in the distribution by economic activity (Fig 6). The results suggest that for Colombia, Honduras, the Dominican Republic, and Mexico, which are the countries with the greatest sensitivity to these changes, the effect of sectoral composition is greater than 20 percent of the total effect on the increase in the informality rate.

Given these scenarios, the question arises as to whether it will be possible to cushion the increases in informality in the years ahead and thus reduce the percentage of the vulnerable population in precarious jobs and labor activities that do not have access to the social security system. As illustrated above, social security systems in the region seem to have functioned as an effective social safety net during the economic contraction resulting from COVID-19, which makes them even more attractive as a means to increase the level of welfare of the population in the new post-2020 context.

### Policy simulations

Taking into consideration the potential negative economic effects of the pandemic on labor market dynamics in Latin America, this section presents estimates of the potential effect of short-term interventions such as exemption from taxes and social security contributions, and long-term measures such as increased schooling. These are the policy scenarios considered in the analysis:

- **Scenario 1** simulates an increase in human capital, measured through the average years of schooling of the population. This scenario assumes an increase of one year of education for the employed population aged 15 and over.
- **Scenario 2** evaluates the future exemption or rescheduling of payments of income tax (*Impuesto sobre la renta*—ISR) for formal jobs using the median income tax rates for individuals reported by the Inter-American Center for Tax Administration (CIAT)–which collects data for tax rates in each country. In this scenario, exempting formal individuals from paying income tax increases their average net market wage, which reflects the average wage of those employed in the formal and informal sectors. Alternatively, it is assumed that the increased liquidity would be used to generate new formal jobs with remuneration equivalent to the average monthly wage.
- **Scenario 3** estimates the effect of exempting or postponing the payment of social security contributions for employers and workers in the formal sector. For each country, the actual rates for employees and employers are used in the simulation. Similar to the previous scenario, it is assumed under Scenario 3 that this exemption from the payment of social security contributions either increases the average market wage, or that the greater implicit liquidity for companies is used to generate new formal jobs with remuneration equivalent to the average monthly wage.

Different studies have documented the various interventions in different countries in the region to mitigate possible increases in informality [13, 54, 62]. They show that, in particular, policies in the same spirit as those considered in Scenarios 2 and 3 have been implemented in some Latin American countries during the pandemic. In terms of the short-term interventions, at least 10 countries in the Latin American region enacted social security weavers, or made labor regulations adjustments as a response measure during the pandemic [6, 13, 52]. The S10 Table contains the parameters used in the simulations. The results of the scenarios are contrasted with the estimated effect of the drop in income associated with the economic crisis resulting from COVID-19 and interpreted as a potential "dampening" effect on the increase in the informality rate.

**Fig 7:**
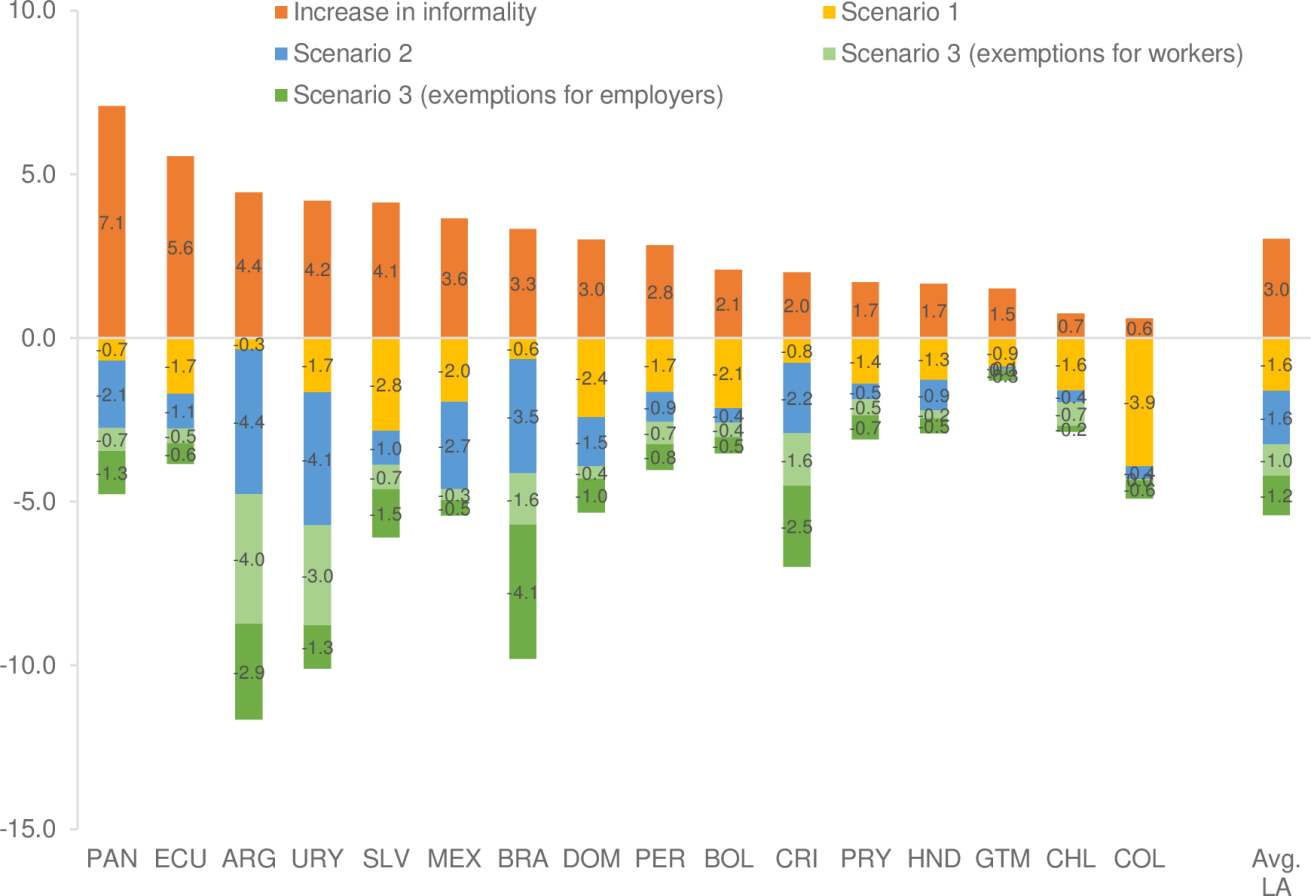
Latin America: Results of the simulation of interventions to cushion impacts on the increase in the informality rate (percentage points). Source: Estimates from household or employment surveys: Argentina—EPH (2019), Bolivia—ECH (2018), Brazil—PNADC (2018), Chile—CASEN (2017), Colombia—GEIH (2018), Costa Rica—ENAHO (2018), Ecuador—ENEMDU (2018), El Salvador—EHPM (2019), Guatemala—ENEI (2018), Honduras—EPHPM—(2018), Mexico—ENIGH (2018), Panama—EPM (2017), Paraguay—EPHC (2018), Peru—ENAHO (2018), Dominican Republic—ENCFT (2017), Uruguay—ECH (2019). p.p.: percentage points. The EPH survey in Argentina only has urban coverage.

Fig 7 presents the results and shows that, on average, an increase of one year of education is associated with a 1.6 percentage point reduction in the informality rate, although with important differences between countries. For example, Colombia, El Salvador, Bolivia, and the Dominican Republic report a greater dampening effect, while Argentina, Brazil, and Costa Rica report smaller reductions.

For Scenario 2, the average effect of the exemption of income tax payments in the region is a 1.6 percentage point reduction in the informality rate, with a greater dampening effect in Argentina, Brazil, and Uruguay, and to a lesser extent in Guatemala, Chile, Colombia, and Bolivia. Scenario 3 considers the exemption from payment of social security contributions for workers and employers simultaneously and separately. The results show that, on average, the dampening effect of the exemptions for both employers and workers reduces the informality rate by 2.3 percentage points, with higher values for Argentina, Brazil, Uruguay, and Costa Rica.

**Fig 8:**
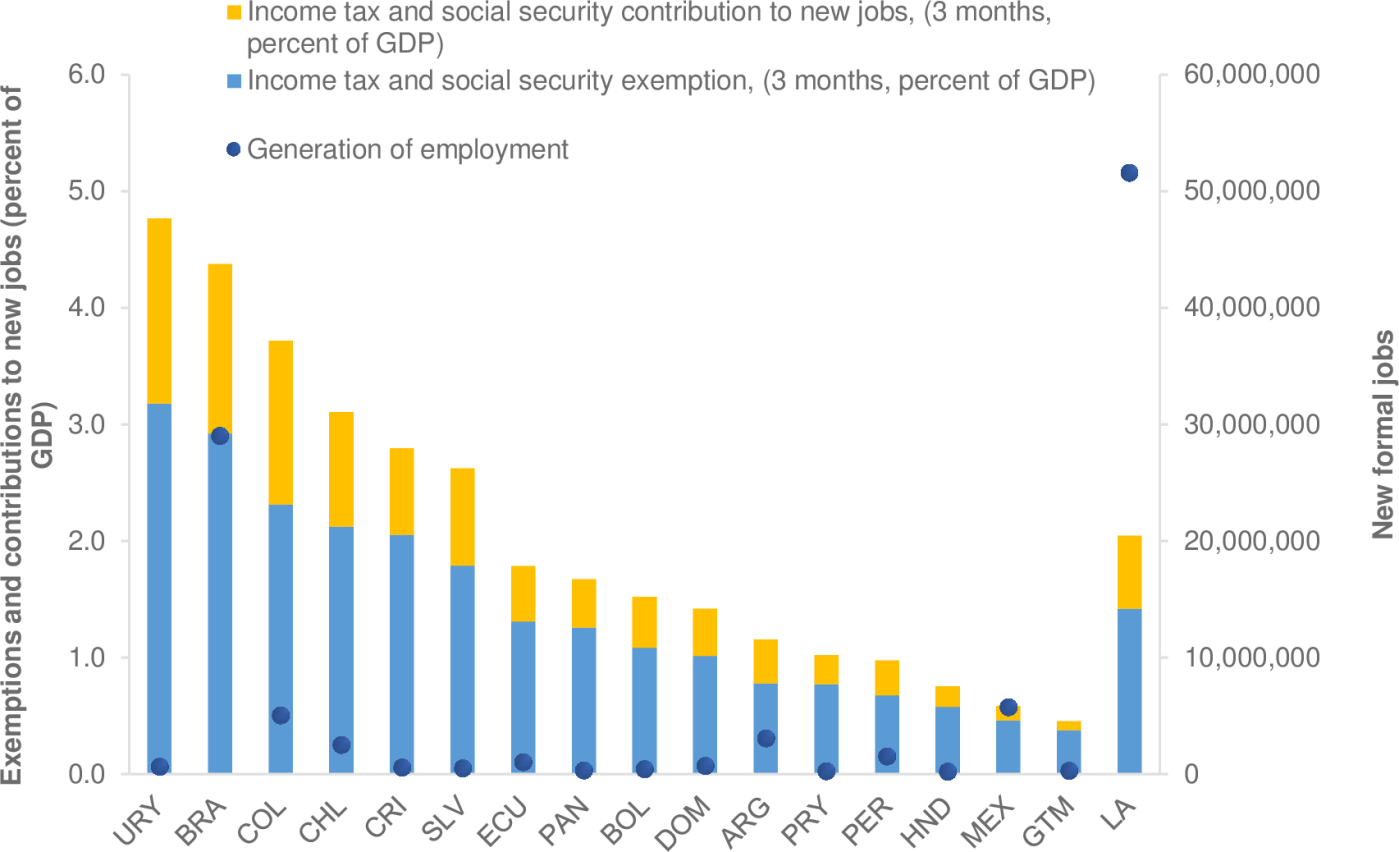
Latin America: Results of the simulation of interventions to cushion the impact of the increase in the informality rate. Source: Estimates from household or employment surveys: Argentina—EPH (2019), Bolivia—ECH (2018), Brazil—PNADC (2018), Chile—CASEN (2017), Colombia—GEIH (2018), Costa Rica—ENAHO (2018), Ecuador—ENEMDU (2018), El Salvador—EHPM (2019), Guatemala—ENEI (2018), Honduras—EPHPM—(2018), Mexico—ENIGH (2018), Panama—EPM (2017), Paraguay—EPHC (2018), Peru—ENAHO (2018), Dominican Republic ENCFT (2017), Uruguay—ECH (2019). The EPH survey in Argentina only has urban coverage.

Finally, Fig 8 presents an approximation of the jobs that could be generated if the greater liquidity obtained as a result of the exemptions from income tax and social security contributions were used to generate new formal jobs, assuming that the remuneration would be equivalent to the average monthly salary identified in the surveys. Fig 8 also reports the approximate cost of the amount of income tax exemptions and social security contributions as a percentage of GDP in each country, assuming a three-month period, as well as the cost of formality for new jobs, measured as access to social security.

On average for the region, the cost of exemptions from income tax and social security contributions for existing jobs represents 1.42 percent of GDP. For Argentina, Chile, Brazil, and Colombia, the cost is more than 3 percent of GDP, while in Guatemala, Honduras, and Peru it is less than 1 percent. For new jobs generated by the greater liquidity inherent in policy actions, the regional average cost is 0.63 percent of GDP, although in the case of Brazil, Colombia, and Uruguay the percentage represents more than 1.4 percent of GDP.

The average total cost, considering existing jobs and additional jobs generated, is 2.04 percent of GDP (blue and yellow bars in Fig 8). Given that there is an inverse relationship between labor income and the probability of being formally employed, the benefits would tend to be concentrated to a greater extent in the region’s middle classes and, above all, in the so-called consolidated middle classes, which are specifically characterized by, among other things, their higher rates of labor formality. This suggests that such policies may have either a progressive or neutral effect on income distribution, although it should also be noted that given the limited fiscal space in the region (which could persist for several years), tax and contribution forgiveness could affect other areas of government spending. Investing in education would require an additional fiscal effort. These resources could be obtained by taxing non-vulnerable households in the informal economy or seeking efficiencies in spending, among other measures.

This result is of greater interest in the context of the set of policies and actions implemented around COVID-19. For example, a recent study presents a similar analysis focusing on estimating the effects on poverty and the social composition of the population in the region, and identify policy options to reverse the negative effects, especially at the bottom of the distribution [9]. This study estimates that the cost of making a six-month transfer to the unemployed population, a transfer to the self-employed, or doubling the benefits of existing social programs (also for a six-month period) would have an average cost in Latin America equivalent to 0.96, 4.29, and 1.46 percentage points of GDP, respectively. The estimated cost of 2.04 percent of GDP of Scenarios 1 and 2 reported in Fig 8 is therefore within the range of such interventions, although presumably with greater benefits for the population located in the middle of the income distribution.

It is important to note, however, that the scenarios are created for the general working population, without distinction by gender or age. A topic of interest for future research is to identify specific buffering policies for women and youth, who as shown the description of the labor dynamics in the region have been most affected during the pandemic. Policies such as support for access to childcare services, online training schemes, access to microcredit for entrepreneurship, or job search assistance mechanisms for youth could be explored as options in this regard.

## Conclusions

This paper has estimated the possible impact of the economic contraction associated with COVID-19 on the labor informality rate for 16 Latin American countries. Preliminary data for 2020 show a substantial contraction in employment and informal employment, while the formal sector seems to have functioned as a safety net for workers with access to contributory social security. The stability of formal jobs may be related to mandatory severance pay, which introduces an element of inflexibility for formal employers. This is combined with the fact that since the beginning of the pandemic its duration has been uncertain, which complicates the determination of the cost-benefit ratio of hiring or firing workers.

To estimate possible scenarios of change in the informality rate in Latin America in the years ahead, the paper has used two approaches. The first is panel data estimation using data constructed from historical labor market figures for 16 countries in the region. This estimation projects the trajectory of the informality rate–assuming a fall in the average wages associated with the contraction of economic activity–for 2021 to 2023. The results of this exercise suggest that the informality rate in the post-pandemic period will increase in the medium term, reaching a level 2.3 percentage points higher than in 2019. The speed of the increase in informality would depend largely on the time it takes to achieve mass vaccination in each country.

Under the survey data estimation, changes in the informality rate associated with drops in labor income are simulated using probit models. The results predict an increase of 3 percentage points in the average informality rate in the region, equivalent to 7.5 million people. In the same sense, eventual changes in the sectoral pattern of the economic recovery would have the potential to increase the informality rate by at least 0.5 additional points.

Finally, to explore strategies to cushion the economic impact of the pandemic on informal employment, this paper has simulated the effect of three policy options. The first consists of increasing the years of education of the labor force (medium-long-term option), which has a buffering capacity of about 50 percent of the predicted increase in informality. The second option simulates the exemption and/or postponement of income tax payments, while the third option analyzes the exemption and/or postponement of social security contributions. According to the estimates, the exemption and/or postponement of social security contributions could cushion between 50 and 75 percent of the expected impact, with an average cost equivalent to 2.04 percent of GDP. Areas of interest for a future research agenda include specific cushioning policies for women and the young population, which seem to have been most affected during the last year.

## Supporting information

S1 Table Sources of household surveys. (PDF)

S2 Table Employment profile in Latin America, circa 2018 (percent). (PDF)

S3 Table Profile of the unemployed and inactive population in Latin America (percent). (PDF)

S4 Table Percentage of the working-age population employed in the formal sector in Latin America. (PDF)

S5 Table Percentage of the working-age population employed in the informal sector in Latin America. (PDF)

S6 Table Percentage of the working-age population that is unemployed Latin America. (PDF)

S7 Table Percentage of the working-age population in a status of inactivity Latin America. (PDF)

S8 Table Latin America: Estimated coefficients of the probit model of the probability of being informal, population aged 15 and older. (PDF)

S9 Table Latin America: Marginal effects of the probability of being informal, population aged 15 years and older. (PDF)

S10 Table Parameters for simulations (percent). (PDF)
