# Supplementary material for: Informality in the time of COVID-19 in Latin America: Implications and policy options
Source: PLoS One. 2021 Dec 16;16(12):e0261277. doi: 10.1371/journal.pone.0261277 (PMC8675676; doi:10.1371/journal.pone.0261277)
Supplement: S1 Table — (PDF) [file pone.0261277.s001.pdf]

S1 Table. Sources of Household Surveys.

Countries	Source of Information	Available at:
Argentina	Permanent Household Survey (Urban) 2019, 2020:Q1, Q2	https://www.indec.gob.ar/indec/web/Institucional-Indec-BasesDeDatos
Bolivia	Household Survey 2018, 2020:Q1, Q2	http://anda.ine.gob.bo/index.php/catalog/84
Brazil	Continuous National Sample Survey 2018, 2020:Q1, Q2, Q3	https://www.ibge.gov.br/estatisticas/sociais/trabalho/9171-pesquisa-nacional-por-amostra-de-domicilios-continua-mensal.html?=&t=microdados
Chile	National Socioeconomic Characterization Survey, 2017 National Employment Survey,<br>JFM 2020,<br>AMJ2020,<br>JAS, 2020	https://www.ine.cl/estadisticas/sociales/mercado-laboral/ocupacion-y-desocupacion
Colombia	Large Integrated Household Survey 2018, February, June, September 2020	https://www.datos.gov.co/Estad-sticas-Nacionales/Gran-Encuesta-Integrada-de-Hogares-GEIH/mcpt-3dws
Costa Rica	National Household Survey 2018	https://www.inec.cr/encuestas/encuesta-nacional-de-hogares
Ecuador	National Employment Survey Unemployment and Underemployment 2018, 2019, 2020:Q2, Q3	https://www.ecuadorencifras.gob.ec/enemdu-2020/
El Salvador	Household Multipurpose Household Survey 2019	https://microdatos.iadb.org/node/11
Guatemala	National Survey of Employment and Income 2018	https://www.ine.gob.gt/estadisticasine/index.php/usuario/enei_menu
Honduras	Permanent Multipurpose Household Survey 2018	https://www.ine.gob.hn/V3/ephpm/
Mexico	National Household Income and Expenditure Survey 2018,<br>National Survey of Occupation and Employment, 2019, 2020:Q1, Q3<br>Telephone Occupation and Employment Survey, 2020:Q2	https://www.inegi.org.mx/programas/enoe/15ymas/
Panama	Permanent Household Survey 2017	https://microdatos.iadb.org/node/11
Paraguay	Continuous Permanent Household Survey 2018, 2020:Q1, Q2, Q3	https://www.ine.gov.py/microdatos/Encuesta-Permanente-de-Hogares-Continua.php
Peru	National Household Survey 2018, 2020:Q1, Q2, Q3	http://iinei.inei.gob.pe/microdatos/
Dominican Republic	2017 Continuing National Labor Force Survey	https://microdatos.iadb.org/node/11
Uruguay	Continuous Household Survey 2019	https://www.ine.gub.uy/encuesta-continua-de-hogares1
IDB Database	Labor Markets and Social Security Information System (SIMS) database	https://www.iadb.org/en/sector/social-investment/sims/sims-institutions
