# Supplementary material for: Informality in the time of COVID-19 in Latin America: Implications and policy options
Source: PLoS One. 2021 Dec 16;16(12):e0261277. doi: 10.1371/journal.pone.0261277 (PMC8675676; doi:10.1371/journal.pone.0261277)
Supplement: S2 Table — (PDF) [file pone.0261277.s002.pdf]

S2 Table. Employment Profile in Latin America, circa 2018 (percent).

Indicator	ARG[a]	BOL	BRA	CHL	COL	CRI	ECU	SLV	GTM	HND	MEX	PAN	PRY	PER	DOM	URY	Latin American Average[c]
Employment rate (>15 years)	55.2	66.6	56.4	55.7	64.0	54.5	64.6	57.7	60.6	63.7	59.4	62.4	68.7	69.3	59.7	58.9	61.1
Women	45.9	55.7	47.1	45.3	51.0	41.5	52.0	43.6	38.8	47.9	44.6	49.4	56.0	61.4	45.7	50.8	48.6
Men	65.7	78.3	66.7	67.7	77.8	68.7	77.7	74.7	85.6	81.6	75.9	76.2	81.6	77.8	74.7	67.7	74.9
Urban		61.2	57.7	56.7	63.3	55.8	61.5	59.8	63.0	62.6	60.2	62.0	68.1	66.8	59.9	59.1	61.2
Rural		79.8	48.5	49.0	66.5	50.9	71.7	54.7	58.4	65.2	58.7	63.6	69.8	78.8	58.8	57.6	62.1
15 - 24	31.4	39.7	43.9	30.6	48.0	35.4	41.0	43.2	53.7	53.6	44.9	42.1	53.6	48.3	40.4	37.8	43.0
25 - 49	75.7	78.9	73.5	75.3	78.2	72.6	79.6	72.1	69.1	73.7	73.4	79.3	80.8	81.5	75.3	83.0	76.4
50 - 64	66.2	78.8	55.3	66.0	67.8	59.3	74.3	62.8	62.7	68.7	61.7	71.1	73.0	79.5	65.7	68.3	67.6
Informality rate (percent of total employment)[b]	47.9	80.5	36.7	31.9	64.0	29.7	63.5	64.6	81.6	82.4	62.1	48.5	77.0	79.7	59.9	24.4	58.4
Women	46.8	82.4	35.2	33.5	63.0	34.8	65.4	67.4	82.6	81.0	62.0	46.5	77.4	82.7	54.7	23.0	58.7
Men	48.8	79.1	37.8	30.6	64.6	26.4	62.1	62.7	81.1	83.2	62.2	49.8	76.8	77.1	63.4	25.6	58.2
Urban		73.8	33.5	30.5	58.0	27.6	58.1	55.5	73.7	94.7	51.2	38.7	70.2	74.6	57.4	23.2	54.7
Rural		93.0	60.2	42.8	85.3	36.4	74.1	79.3	89.3	72.7	72.0	72.2	88.8	96.3	70.4	31.2	70.9
15 - 24	65.5	93.2	47.1	35.9	72.1	36.2	79.0	73.3	89.0	87.1	65.7	56.9	84.7	85.2	60.8	37.6	66.8
25 - 49	42.0	75.7	32.5	25.9	56.9	25.4	58.7	59.6	75.5	77.4	56.1	41.4	71.3	75.3	55.9	20.2	53.1
50 - 64	48.8	80.4	36.9	34.6	69.3	30.0	59.7	65.3	85.6	86.0	68.4	50.6	82.6	79.5	66.7	23.3	60.5
Percentage of people working in companies with 1 to 5 workers (percent of total employment)	47.6	80.6	73.8	31.6	63.1	44.5	82.6	61.5	63.3	45.9	52.3	43.8	56.3	74.8	56.7	40.9	57.5
Women	45.3	83.3	74.8	35.6	61.5	48.8	86.5	66.5	71.0	35.1	54.1	43.4	52.4	80.3	47.9	41.7	58.0
Men	49.2	78.5	73.3	28.6	64.3	41.7	79.8	58.4	59.2	58.1	51.1	44.1	59.0	70.0	61.7	40.2	57.3
Urban		75.0	72.9	30.6	57.4	41.0	82.6	56.5	57.2	37.7	42.0	35.3	46.3	69.9	54.1	37.9	53.1
Rural		89.2	87.8	39.9	83.7	55.3	86.4	69.7	69.3	56.8	61.6	64.6	73.6	89.4	67.4	56.8	70.1
15 - 24	54.7	74.8	76.3	22.3	63.1	36.2	76.1	59.9	62.2	39.1	49.4	42.0	51.5	64.6	56.2	40.6	54.3
25 - 49	43.1	76.6	75.2	26.1	57.1	39.4	79.8	56.2	58.5	48.8	46.9	38.2	51.0	69.6	52.7	36.6	53.5
50 - 64	50.5	86.9	73.0	40.0	72.6	55.5	88.4	69.2	73.6	54.9	61.0	49.2	70.4	83.1	63.4	46.0	64.9

Sources: Estimates from household or employment surveys: Argentina - EPH (2019), Bolivia - ECH (2018), Brazil - PNADC (2018), Chile - CASEN (2017), Colombia - GEIH (2018), Costa Rica - ENAHO (2018), Ecuador - ENEMDU (2018), El Salvador - EHPM (2019), Guatemala - ENEI (2018), Honduras - EPHPM - (2018), Mexico - ENIGH (2018), Panama - EPM (2017), Paraguay - EPHC (2018), Peru - ENAHO (2018), Dominican Republic - ENCFT (2017), Uruguay - ECH (2019).

[a] The EPH survey in Argentina only has urban coverage.

[b] Formality is defined as having access to social security.

[c] Simple average for Latin America and the Caribbean.
