# Supplementary material for: Informality in the time of COVID-19 in Latin America: Implications and policy options
Source: PLoS One. 2021 Dec 16;16(12):e0261277. doi: 10.1371/journal.pone.0261277 (PMC8675676; doi:10.1371/journal.pone.0261277)
Supplement: S3 Table — (PDF) [file pone.0261277.s003.pdf]

S3 Table. Profile of the Unemployed and Inactive Population in Latin America (percent).

Indicator	ARG[a]	BOL	BRA	CHL	COL	CRI	ECU	SLV	GTM	HND	MEX	PAN	PRY	PER	DOM	URY	Latin American Average[b]
Unemployment rate (>15 years)	6.6	2.3	7.6	4.7	6.9	4.9	2.0	2.4	3.0	4.0	2.1	3.7	4.5	2.5	3.3	5.7	4.3
Women	5.8	2.5	7.4	4.4	7.9	4.5	1.9	1.9	3.7	3.9	1.6	3.7	4.4	2.5	3.7	6.1	4.4
Men	7.4	2.0	7.7	5.2	5.9	5.3	2.1	3.1	2.2	4.0	2.6	3.7	4.6	2.6	2.8	5.3	4.3
Urban		3.0	8.0	5.0	7.9	5.0	2.6	2.8	3.0	5.5	2.5	4.3	5.2	3.0	3.6	6.0	4.7
Rural		0.5	5.0	3.3	3.5	4.6	0.7	1.9	3.0	1.9	1.7	2.3	3.2	0.6	1.9	4.2	2.8
15 - 24	11.4	3.4	16.3	8.2	12.0	9.1	3.2	4.5	4.6	6.8	3.4	7.5	8.8	5.7	5.6	14.5	8.5
25 - 49	7.6	2.7	8.1	5.9	7.3	5.8	2.4	2.5	2.6	3.8	2.3	4.0	3.8	2.3	3.9	5.7	4.5
50 - 64	4.5	1.1	3.6	3.2	4.4	2.2	0.9	0.9	2.1	1.7	1.3	1.3	2.2	1.3	1.1	2.7	2.3
Inactivity rate (>15 years)	38.2	31.1	36.0	39.6	30.0	40.6	33.4	39.8	36.8	32.3	38.5	33.9	26.8	28.2	36.3	35.4	34.8
Women	48.3	41.8	45.5	50.4	41.6	54.0	46.0	54.5	58.0	48.2	53.8	46.9	39.6	36.1	49.7	43.1	47.3
Men	26.8	19.7	25.6	27.1	17.6	26.0	20.2	22.2	12.5	14.4	21.5	20.1	13.8	19.6	21.9	26.9	21.0
Urban		35.8	34.4	38.4	29.5	39.2	35.9	37.4	34.4	31.9	37.3	33.8	26.7	30.1	35.7	34.9	34.4
Rural		19.7	46.5	47.7	31.7	44.5	27.6	43.4	39.0	32.9	39.6	34.2	27.0	20.7	38.8	38.2	35.4
15 - 24	57.2	56.9	39.7	61.2	42.8	55.5	55.8	52.3	42.4	39.5	51.7	50.4	37.5	46.0	52.5	47.7	49.3
25 - 49	16.7	18.4	18.5	18.8	15.0	21.6	18.0	25.4	28.7	22.5	24.3	16.7	15.5	16.2	20.0	11.3	19.2
50 - 64	29.3	20.1	41.2	30.7	28.1	38.5	24.8	36.3	35.5	29.6	37.0	27.6	24.8	19.1	33.0	29.0	30.3

Sources: Estimates from household or employment surveys: Argentina - EPH (2019), Bolivia - ECH (2018), Brazil - PNADC (2018), Chile - CASEN (2017), Colombia - GEIH (2018), Costa Rica - ENAHO (2018), Ecuador - ENEMDU (2018), El Salvador - EHPM (2019), Guatemala - ENEI (2018), Honduras - EPHPM - (2018), Mexico - ENIGH (2018), Panama - EPM (2017), Paraguay - EPHC (2018), Peru - ENAHO (2018), Dominican Republic - ENCFT (2017), Uruguay - ECH (2019).

[a] The EPH survey in Argentina only has urban coverage.

[b] Simple average for Latin America and the Caribbean.
