# Supplementary material for: Informality in the time of COVID-19 in Latin America: Implications and policy options
Source: PLoS One. 2021 Dec 16;16(12):e0261277. doi: 10.1371/journal.pone.0261277 (PMC8675676; doi:10.1371/journal.pone.0261277)
Supplement: S6 Table — (PDF) [file pone.0261277.s006.pdf]

S6 Table. Percentage of the Working-age Population That Is Unemployed Latin America.

Country	2006	2007	2008	2009	2010	2011	2012	2013	2014	2015	2016	2017	2018	2019
Argentina[a]	6.5	5.3	5.2	6.0	5.0	4.9	5.1	4.6	4.9	4.6	5.5	5.4	6.3	6.6
Bolivia	3.5	3.8	2.0	2.2		1.7	1.5	1.8	1.6	2.3	2.4	2.4	2.4	
Brazil	6.3	6.1	5.4	6.3		4.9	4.5	4.7	5.1	7.0	8.3	9.1	8.8	8.6
Chile	4.7			6.5		5.0		4.7		5.1		5.5		
Colombia	9.7	8.6	8.0	8.8	8.5	7.8	7.7	7.2	6.8	6.9	6.9	7.2	7.2	
Costa Rica	3.9	3.1	3.3	5.2	4.7	5.1	5.2	5.7	5.7	5.7	5.3	4.9	5.5	6.1
Ecuador	2.7	2.3	2.9	3.3	2.5	2.1	2.3	2.1	2.1	2.6	3.0	2.8	2.2	2.5
El Salvador	2.3	2.4	2.5	3.2	3.1	2.7	2.5	2.4	2.7	2.5	2.8	2.8	2.6	2.7
Guatemala	0.7				1.5	1.8	1.4	1.4	1.3	1.3	1.4	1.2	1.4	0.8
Honduras	2.1	2.3	2.5	2.7	3.5	3.8	3.1	3.9	4.9	4.2	4.6	3.9	2.2	
Mexico	2.3		3.0		3.7		3.0		3.2		2.2		2.1	
Panama	5.4	4.1	3.7	3.8	3.4	3.7	3.1	2.9	2.9	3.8	4.2	4.1	4.4	4.8
Paraguay	4.9	4.2	4.2	4.9	3.2	4.1	3.7	3.8	4.5	4.0	4.6	3.9	4.3	
Peru	3.3	3.6	3.5	3.5	3.2	3.0	2.8	2.9	2.6	2.5	3.0	3.0	2.9	2.9
Dominican Republic	3.4	3.1	3.0	3.5	3.1	3.9	4.6	4.7	3.9	3.9	3.5	4.0	4.6	
Uruguay	8.0	7.2	6.2	6.0	5.5	5.0	5.0	5.0	5.1	5.8	6.1	6.1	6.4	6.8
Average for Latin America[b]	4.4	4.3	3.9	4.7	3.9	4.0	3.7	3.8	3.8	4.2	4.3	4.4	4.2	4.6

Source: Estimates from the IDB's Labor Markets and Social Security Information System (SIMS) database, 2020.
[a] The EPH survey in Argentina only has urban coverage.
[b] Simple average for Latin America.
