# Supplementary material for: Informality in the time of COVID-19 in Latin America: Implications and policy options
Source: PLoS One. 2021 Dec 16;16(12):e0261277. doi: 10.1371/journal.pone.0261277 (PMC8675676; doi:10.1371/journal.pone.0261277)
Supplement: S7 Table — (PDF) [file pone.0261277.s007.pdf]

S7 Table. Percentage of the Working-age Population in a Status of Inactivity Latin America.

Country	2006	2007	2008	2009	2010	2011	2012	2013	2014	2015	2016	2017	2018	2019
Argentina[a]	31.2	32.4	32.2	31.9	32.5	31.7	31.5	32.6	33.4	33.8	33.0	31.8	31.4	30.8
Bolivia	25.6	27.7	26.6	26.2		26.0	29.3	28.6	26.6	31.1	27.2	30.0	30.2	
Brazil	26.2	26.5	26.3	26.1		28.2	28.3	28.6	27.4	28.6	30.0	29.5	29.6	28.7
Chile	37.4			37.8		37.6		35.8		34.3		32.6		
Colombia	31.9	31.5	31.5	29.0	27.5	27.1	26.2	25.9	25.9	25.6	26.1	26.0	26.1	
Costa Rica	35.1	34.1	34.6	34.7	35.9	34.1	34.4	34.2	34.6	34.1	33.2	35.1	34.9	35.5
Ecuador	29.0	30.3	32.0	32.4	34.4	34.5	34.7	35.2	33.2	32.1	30.6	29.8	31.2	31.3
El Salvador	37.9	36.9	36.4	36.9	37.6	37.5	36.7	36.4	37.2	37.8	37.2	37.2	37.5	36.4
Guatemala	42.3				45.5	42.7	40.9	41.9	38.2	37.2	38.0	38.4	37.9	39.5
Honduras	38.0	37.9	39.1	36.8	35.1	37.3	38.4	34.9	33.4	33.6	33.8	33.3	33.0	
Mexico	32.4		34.6		35.2		31.3		33.5		30.7		30.5	
Panama	33.5	32.9	31.6	31.6	32.5	33.1	31.6	33.3	31.0	29.7	29.5	29.4	27.6	27.9
Paraguay	28.6	27.6	27.5	26.1	28.7	27.8	24.9	25.9	27.4	27.3	26.0	26.1	24.7	
Peru	26.0	24.5	25.1	23.2	23.2	24.7	25.0	25.0	25.8	26.3	25.5	25.2	25.3	25.0
Dominican Republic	39.4	39.3	39.2	40.9	39.4	37.8	36.9	37.0	37.0	35.8	35.8	33.8	30.9	
Uruguay	26.6	25.1	24.9	24.2	24.7	23.5	24.0	24.4	23.7	24.6	24.8	25.2	25.7	25.7
Average for Latin America[b]	32.6	31.3	31.5	31.3	33.2	32.2	31.6	32.0	31.2	31.5	30.8	30.9	30.4	31.2

Source: Estimates from the IDB's Labor Markets and Social Security Information System (SIMS) database, 2020.
[a] The EPH survey in Argentina only has urban coverage. [b] Simple average for Latin America.
