# Supplementary material for: Informality in the time of COVID-19 in Latin America: Implications and policy options
Source: PLoS One. 2021 Dec 16;16(12):e0261277. doi: 10.1371/journal.pone.0261277 (PMC8675676; doi:10.1371/journal.pone.0261277)
Supplement: S9 Table — (PDF) [file pone.0261277.s009.pdf]

S9 Table. Latin America: Marginal Effects of the Probability of Being Informal, Population Aged 15 years and Older.

Variables	(1)	(2)	(3)	(4)	(5)	(6)	(7)	(8)	(9)	(10)	(11)	(12)	(13)	(14)	(15)	(16)
	ARG[a]	BOL	BRA	CHL	COL	CRI	DOM	ECU	GTM	MEX[b]	HND	PAN	PER	PRY[c]	SLV	URY
Ln(Monetary labor income)	-0.264***	-0.0905***	-0.216***	-0.0346***	-0.0375***	-0.178***	-0.143***	-0.177***	-0.156***	-0.209***	-0.142***	-0.207***	-0.129***	-0.135***	-0.175***	-0.172***
	(0.00660)	(0.00993)	(0.00205)	(0.00132)	(0.000847)	(0.00387)	(0.00768)	(0.00619)	(0.00667)	(0.00318)	(0.00628)	(0.00940)	(0.00283)	(0.00467)	(0.00636)	(0.00182)
Sex (Male=1)	0.0351***	-0.0226***	0.0333***	-0.0500***	-0.0693***	-0.0409***	-0.00977	0.0217**	-0.0275***	0.00613	0.00916	0.0228**	-0.00540	-0.0178***	-0.0507***	0.0518***
	(0.0104)	(0.00729)	(0.00310)	(0.00470)	(0.00271)	(0.00738)	(0.0126)	(0.00882)	(0.00778)	(0.00413)	(0.00830)	(0.00965)	(0.00474)	(0.00639)	(0.00779)	(0.00361)
Rural		-0.0235**	0.0129***	0.0625***	0.105***	-0.00275	-0.00913	-0.0468***	0.00373	0.0540***	0.0298***	0.0243***	0.0661***	0.0289***	0.0510***	0.00248
		(0.0103)	(0.00355)	(0.00588)	(0.00508)	(0.00753)	(0.0122)	(0.00992)	(0.00835)	(0.00366)	(0.00925)	(0.00844)	(0.00527)	(0.00730)	(0.00680)	(0.00430)
25 to 49 years old	-0.0267*	-0.0790***	-0.0130***	-0.0455***	-0.106***	-0.0397***	0.000866	-0.0865***	-0.0222**	-0.00276	-0.0169*	-0.000290	-0.00322	-0.0418***	-0.0481***	-0.00539
	(0.0150)	(0.00925)	(0.00383)	(0.00748)	(0.00349)	(0.00955)	(0.0142)	(0.0110)	(0.00880)	(0.00474)	(0.00880)	(0.0107)	(0.00655)	(0.00782)	(0.00873)	(0.00483)
50 to 64 years old	0.0409**	-0.130***	0.00132	-0.000602	-0.0522***	-0.0497***	0.0451**	-0.131***	0.0194	0.0430***	0.00889	0.0529***	0.00651	-0.00105	-0.0623***	0.00206
	(0.0172)	(0.0137)	(0.00483)	(0.00796)	(0.00423)	(0.0111)	(0.0177)	(0.0130)	(0.0123)	(0.00595)	(0.0131)	(0.0130)	(0.00732)	(0.0102)	(0.0125)	(0.00543)
65 and over	0.193***	-0.137***	0.243***	0.285***	0.189***	0.235***	0.0570*	-0.115***	0.0511**	0.157***	0.0391	0.308***	0.0718***	0.101***	-0.0497**	0.157***
	(0.0292)	(0.0215)	(0.00971)	(0.0113)	(0.00711)	(0.0266)	(0.0296)	(0.0174)	(0.0239)	(0.0103)	(0.0240)	(0.0253)	(0.0111)	(0.0151)	(0.0227)	(0.00951)
Average years of education	-0.00337**	-0.0194***	-0.00639***	-0.0158***	-0.0326***	-0.00753***	-0.0220***	-0.0154***	-0.00832***	-0.0180***	-0.0119***	-0.00667***	-0.0157***	-0.0134***	-0.0245***	-0.0170***
	(0.00135)	(0.000971)	(0.000405)	(0.000622)	(0.000305)	(0.000944)	(0.00135)	(0.000914)	(0.000951)	(0.000485)	(0.00109)	(0.00146)	(0.000612)	(0.00135)	(0.000842)	(0.000511)
Mining and quarrying	-0.387***	-0.247***	-0.144***	-0.227***	-0.257***	0.0205	-0.464***	-0.238***	-0.0324	-0.441***	-0.0179	-0.266***	-0.154***		-0.0276	-0.0344
	-0.0786	(0.0224)	(0.0187)	(0.0189)	(0.0171)	(0.115)	(0.118)	(0.0489)	(0.0603)	(0.0256)	(0.0795)	(0.0771)	(0.0150)		(0.112)	(0.0488)
Manufacturing industry	-0.0964**	-0.0969***	-0.124***	-0.0199**	-0.125***	0.00345	-0.220***	0.00224	-0.0690***	-0.302***	-0.141***	-0.0721***	-0.0609***	-0.208***	-0.271***	0.0457***
	(0.0463)	(0.0165)	(0.00616)	(0.00878)	(0.00669)	(0.0144)	(0.0263)	(0.0156)	(0.0129)	(0.00758)	(0.0162)	(0.0182)	(0.00859)	(0.0147)	(0.0145)	(0.00717)
Electricity, gas, and water	-0.303***	-0.330***	-0.146***	-0.246***	-0.510***	-0.0964***	-0.717***	-0.316***	-0.0527		-0.110***	-0.237***	-0.106***	-0.390***	-0.263***	-0.0922***
	(0.0628)	(0.0414)	(0.0145)	(0.0243)	(0.0260)	(0.0349)	(0.0515)	(0.0553)	(0.0358)		(0.0361)	(0.0876)	(0.0388)	(0.0345)	(0.0331)	(0.0191)
Construction	0.102**	0.00922	0.135***	0.0103	-0.0151**	0.134***	0.196***	0.224***	0.126***	0.0597***	0.0684***	-0.00128	0.0794***	0.0601***	-0.0433***	0.172***
	(0.0472)	(0.0176)	(0.00655)	(0.00889)	(0.00758)	(0.0156)	(0.0298)	(0.0169)	(0.0205)	(0.00906)	(0.0206)	(0.0167)	(0.00977)	(0.0196)	(0.0167)	(0.00716)
Retail, restaurants and hotels	-0.0194	-0.000280	-0.0337***	0.0677***	0.0209***	0.0351***	-0.0297	0.126***	0.0286**	-0.0993***	0.00601	-0.109***	0.0463***	-0.114***	-0.103***	0.0610***
	(0.0455)	(0.0167)	(0.00544)	(0.00741)	(0.00648)	(0.0122)	(0.0239)	(0.0133)	(0.0132)	(0.00792)	(0.0170)	(0.0149)	(0.00765)	(0.0141)	(0.0145)	(0.00632)
Transportation and storage	-0.102**	0.0494***	0.000455	0.0282**	-0.0527***	0.0850***	0.212***	0.128***	0.0462**	-0.0603***	0.0514**	0.0636***	0.0760***	-0.169***	-0.106***	-0.0230**
	(0.0476)	(0.0183)	(0.00749)	(0.0115)	(0.00707)	(0.0172)	(0.0309)	(0.0181)	(0.0191)	(0.0106)	(0.0245)	(0.0179)	(0.00932)	(0.0182)	(0.0192)	(0.00943)
Financial and insurance establishments	-0.143***	-0.248***	-0.0438***	-0.0856***	-0.177***	-0.0621**	-0.213***	-0.0144	-0.111***	-0.188***	-0.0967***	-0.222***	-0.148***	-0.155***	-0.343***	-0.0704***
	(0.0506)	(0.0269)	(0.0118)	(0.0103)	(0.00709)	(0.0316)	(0.0357)	(0.0186)	(0.0224)	(0.0105)	(0.0239)	(0.0290)	(0.0175)	(0.0175)	(0.0198)	(0.0156)
Social and community services	-0.198***	-0.207***	-0.0739***	-0.0516***	-0.178***	0.0322***	-0.172***	-0.0940***	-0.103***	-0.126***	-0.0535***	-0.119***	-0.112***	-0.224***	-0.219***	0.0319***
	(0.0450)	(0.0151)	(0.00542)	(0.00763)	(0.00659)	(0.0120)	(0.0243)	(0.0146)	(0.0128)	(0.00827)	(0.0170)	(0.0153)	(0.00724)	(0.0143)	(0.0149)	(0.00617)
Pseudo-R2	0.279	0.387	0.230	0.0970	0.266	0.272	0.219	0.271	0.399	0.254	0.425	0.295	0.345	0.261	0.289	0.370
Observations	20,881	14,612	177,165	85,045	305,850	14,376	8,097	18,767	8,653	125,479	9,044	17,595	60,061	23,877	27,112	48,268

Sources: Estimates from household or employment surveys: Argentina - EPH (2019), Bolivia - ECH (2018), Brazil - PNADC (2018), Chile - CASEN (2017), Colombia - GEIH (2018), Costa Rica - ENAHO (2018), Ecuador - ENEMDU (2018), El Salvador - EHPM (2019), Guatemala - ENEI (2018), Honduras - EPHPM - (2018), Mexico - ENIGH (2018), Panama - EPM (2017), Paraguay - EPHC (2018), Peru - ENAHO (2018), Dominican Republic - ENCFT (2017), Uruguay - ECH (2019).

Note: Standard errors in parentheses. *** p<0.01, ** p<0.05, * p<0.1

[a] The EPH survey in Argentina only covers urban areas.

[b] In Mexico, the manufacturing industry branch includes extractive industries.

[c] In Paraguay, the electricity, gas and water branch is not included.
