# Supplementary material for: Informality in the time of COVID-19 in Latin America: Implications and policy options
Source: PLoS One. 2021 Dec 16;16(12):e0261277. doi: 10.1371/journal.pone.0261277 (PMC8675676; doi:10.1371/journal.pone.0261277)
Supplement: S10 Table — (PDF) [file pone.0261277.s010.pdf]

S10 Table. Parameters for Simulations (percent).

Country	Projected GDP Growth for 2020	Simulation of Income Drop Shock	Median Income Tax Liability	Median of Workers' Social Security Contributions	Median of Employers' Social Security Contributions
Argentina	-10.4	-13.1	19	17	13
Bolivia	-7.9	-23.0	13	13	15
Brazil	-4.5	-12.7	19	9	22
Chile	-6.0	-21.0	14	26	7
Colombia	-7.9	-15.4	24	8	29
Costa Rica	-5.5	-9.1	13	9	14
Ecuador	-11.0	-26.4	15	10	11
El Salvador	-9.0	-20.9	15	12	20
Guatemala	-2.0	-10.4	6	5	11
Honduras	-6.6	-14.0	18	5	9
Mexico	8.5	15.6	21	2	4
Panama	-9.0	-26.2	15	7	11
Paraguay	-4.0	-12.5	9	9	14
Peru	-12.0	-24.0	17	13	15
Dominican Republic	-6.0	-19.7	20	6	14
Uruguay	-4.5	-7.6	25	18	8

Sources: Data from IMF, 2020 ; Inter-American Center of Tax Administration (CIAT), 2018; data from the social security programs for each country; and regional employment and household surveys.
